# Transcriptomic and proteomic analyses uncover the drought adaption landscape of Phoebe zhennan

**DOI:** 10.1186/s12870-022-03474-3

**Published:** 2022-03-03

**Authors:** Na Xie, Bo Li, Jing Yu, Ruxia Shi, Qin Zeng, Yunli Jiang, Dan Zhao

**Affiliations:** 1 Institute of Agro-Bioengineering and College of Life Sciences, The Key Laboratory of Plant Resources Conservation and Germplasm Innovation in Mountainous Region (Ministry of Education), Guizhou University, Guiyang, 550025 Guizhou China; 2 Guizhou Academy of Agricultural Sciences, Guizhou Plant Conservation Technology Center, Guiyang, 550006 Guizhou China; 3 Tobacco Molecular Genetics Key Laboratory of China Tobacco, Guizhou Academy of Tobacco Science, Guiyang, 550081 China; 4 Guizhou Academy of Forestry, Guiyang, 550005 Guizhou China

**Keywords:** *Phoebe zhennan*, Drought, Antioxidant enzyme, Hormone, Transcriptomic, Proteomic

## Abstract

**Background:**

*Phoebe zhennan* S.Lee (nanmu) is listed as a threatened tree species in China, whose growth and development, especially during the seedling stage, can be severely limited by drought. Previous studies on nanmu responses to drought stress involved physiological and biochemical analyses, while the molecular mechanisms remained unclear. Therefore, it is of great significance to carry out molecular biology research on the drought resistance of nanmu and reveal the genetic background and molecular regulation mechanism of nanmu drought resistance.

**Results:**

Drought stress enhanced the soluble sugar (SS), free proline(PRO), superoxide anion (O2·−), and hydrogen peroxide (H_2_O_2_) contents as well as the peroxidase (POD) and monodehydroascorbate reductase (MDHAR) activities of nanmu. However, glutathione S-transferase (GST) activity was sensitive to drought stress. Further transcriptomic and proteomic analyses revealed the abundant members of the differentially expressed genes(DEGs) and differentially expressed proteins(DEPs) that were related to phenylpropanoid and flavonoid biosynthesis, hormone biosynthesis and signal transduction, chlorophyll metabolism, photosynthesis, and oxidation-reduction reaction, which suggested their involvement in the drought response of nanmu. These enhanced the osmotic regulation, detoxification, and enzyme-induced and non-enzyme-induced antioxidant ability of nanmu. Moreover, 52% (447/867) of proteins that were up-regulated and 34% (307/892) down-regulated ones were attributed to the increase and decrease of transcription abundance. Transcript up (T^U^) and protein up (P^U^) groups had 447 overlaps, while transcript down (T^D^) and protein down (P^D^) groups had 307 overlaps, accounting for 54% of up and 35% of down-regulated proteins. The lack of overlap between DEGs and DEPs also suggested that post-transcriptional regulation has a critical role in nanmu response to drought.

**Conclusions:**

Our research results provide significant insights into the regulatory mechanisms of drought stress in nanmu.

**Supplementary Information:**

The online version contains supplementary material available at 10.1186/s12870-022-03474-3.

## Background

Drought can significantly affect the growth and development of plants throughout their whole life cycle [1]. Phoebe zhennan S. Lee (Lauraceae; Golden Phoebe) is a slow-growing forest tree whose precious timber tree species and landscaping tree species are unique to China’s subtropics. Nanmu is an important timber-producing tree species that have been listed as threatened species by the International Union for Conservation of Nature (IUCN) and is therefore nationally protected in China [2]. In addition to human activities, plantation studies have shown that nanmu trees rarely reach their full growth potential due to abiotic stress constraints, such as drought stress. Therefore, improving the understanding of the stress tolerance mechanism of nanmu is beneficial for tree reproduction and cultivation.

Numerous studies have shown that plants develop various strategies to survive and improve their tolerance to drought under water-stressed conditions [3]. The most common strategies are morphological changes, which mainly include leaf curling. Rapid stomatal closing and increased root length are key morphological events that reduce water loss and provide greater access to water, respectively [4, 5]. Meanwhile, a series of complex biochemical and physiological responses occur, which mainly include antioxidant enzymes, endogenous hormones, photosynthetic systems, and osmotic regulators [6]. Plants have evolved an antioxidant defense system that includes enzymatic and non-enzymatic elements present in plant cells to counteract oxidative stress [7, 8]. These elements include accumulation of soluble proteins,SS, O2·−, and H_2_0_2._ in cells [9, 10] as well as activation of reactive oxygen species (ROS), POD, catalase (CAT), and superoxide dismutase (SOD) in cells [11, 12], which are involved in the processes of water preservation and cell membrane stability. Drought has also been identified as the most severe agricultural and environmental issue globally. Plants employ efficient and sophisticated regulatory mechanisms to cope with drought stress where numerous genes and thousands of biochemical and cellular processes coordinate mediate various adaptive processes [13, 14]. When plants are under drought stress, plants activate physiological, biochemical, metabolic, and defense systems by altering gene expression patterns [15, 16].

Furthermore, the hormone signaling pathways have also been identified as critical for drought stress response in plants [17]. Drought accumulates high abscisic acid (ABA) levels via up-regulation of ABA-related biosynthetic genes so as to close stomata and reduce transpiration [18]. In addition, the gene networks involved in the biosynthesis and signaling of auxin, ethylene (ET), jasmonic acid (JA), salicylic acid (SA), flavonoid, Phenylpropanoid, and brassinosteroids (BRs) are also activated under drought stress [19, 20]. Genes related to heat shock proteins (HSPs) secondary metabolism have also been found to significantly contribute to drought stress [21].

Over recent years, the evaluation of the adaptive mechanisms in crops, model plants, and woody plants under drought stress has gained increasing attention [22, 23]. Based on the next-generation sequencing technology, several genes and pathways related to drought stress tolerance have been identified through transcriptomic and genomic techniques [24]. In addition to iTRAQ quantitative proteomic analysis, liquid chromatography-tandem mass spectrometry (LC-MS/MS) technology, and multiplex analysis of plant proteins, plant multi-omics studies under drought conditions, such as *Cassava*, and *Agropyron mongolicum* Keng have also been reported [25, 26]. These studies revealed more genes and protein pathways in response to drought stress and provided important information for systematically elucidating the mechanism network of plants in response to drought at the biological level. At present, there are few studies on the molecular mechanism of drought stress in Lauraceae plants, especially *Phoebe* [27, 28], whose leaves accumulated high levels of osmotic pressure, sugar, and protein, accompanied by high levels of malondialdehyde (MDA), ROS, SOD, CAT, and POD under drought stress for 30 days.

Although this study provides essential and fundamental information about nanmu response to drought, the understanding of global molecular mechanisms underlying drought tolerance is still very limited. Recently, based on the next-generation sequencing technology, the multi-omics analysis has become an efficient and economical tool for the comprehensive elucidation of gene regulatory networks in numerous biological processes. Therefore, in this study, we investigated the physiological and molecular regulation mechanism of nanmu response to drought stress by analyzing the physiological and biochemical parameters. DEGs and DEPs were further identified, including the biological processes such as photosynthesis, carbohydrate metabolism, secondary metabolism, hormones biosynthesis, and signaling. The results of this study significantly further our understanding of the transcriptomic and proteomic mechanisms in drought stress response and growth regulation of nanmu seedlings.

## Results

### Physiological responses of *Phoebe zhennan* (nanmu) under drought stress

**Fig. 1:**
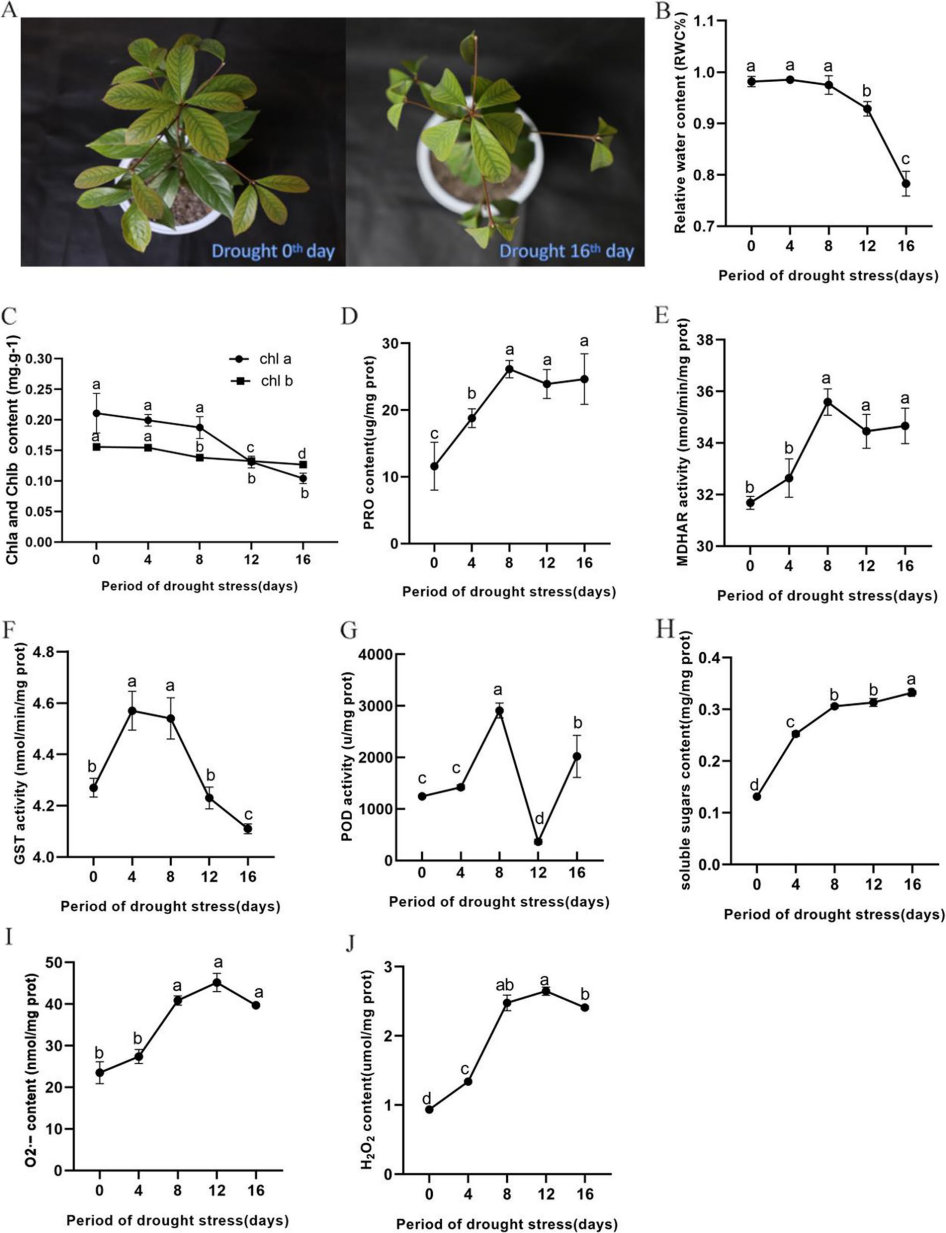
Physiological responses to drought stress of 0–16 days. **A** Nanmu plant at day 16 of water withholding (right) compared to the control plants of the same age (left). **B** Leaf RWC. **C** Chl a and b content. **D** PRO content. **E** MDHAR activity. **F** GST activity. **G** POD activity. **H** Soluble sugars content. **I** O2· − content. **H** H_2_0_2_ content. The different letters indicate significant differences detected by the Tukey LSD test (*P* < 0.05)

To estimate the dynamic of nanmu in response to drought stress, we investigated the morphological changes and physiological responses involved in water content maintenance and oxidative damage in plant cells at five time-points (0, 4, 8, 12, and 16 d) in leaves (Fig. 1). Compared with the control 0-day drought-stressed (0d-DS), nanmu leaves were severely wilted after the 16-day drought-stress (16d-DS) (Fig. 1A). Consistently, the relative water content (RWC) rapidly decreased by 78.27% after 16 d of drought stress (Fig. 1B). The significant reduction of chlorophyll a (Chl a) and chlorophyll (Chlb) during stress was observed at 12 and 8 d, respectively (Fig. 1C). In contrast, PRO and MDHAR content increased during drought stress (Fig. 1D, E). GST activity significantly increased from day 4 onwards and maintained this activity before the gradual decline throughout the remaining course of the experiment (Fig. 1F). The POD activity first increased and then decreased after 8 d of drought stress (Fig. 1G). The SS content of nanmu was found to continuously rise during drought stress (Fig. 1H). The O2· − content of nanmu under drought stress did not significantly change in the early stage. It significantly increased after 8d, after which it finally stabilized (Fig. 1I). The content of H_2_0_2_ increased in the early stage and remained stable in the later stage.

**Fig. 2:**
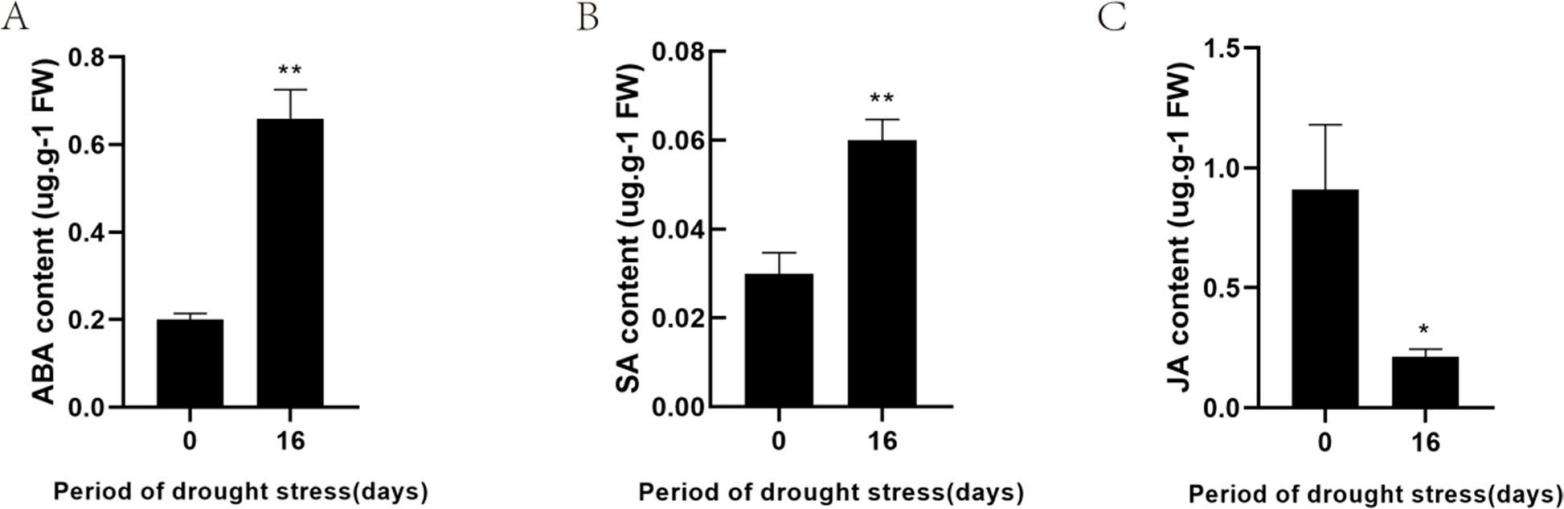
Hormone responses to drought stress. **A** ABA levels measured at 0 and 16d. **B** SA levels measured at 0 and 16d. **C** JA levels measured at 0 and 16d. Asterisks indicate significant differences detected by the Tukey LSD test (*P < 0.05, **P < 0.01)

We also analyzed the changes in ABA, SA, and JA in nanmu leaves under drought stress since these are commonly involved in various stresses. The ABA and SA content in nanmu leaves increased by 228.33 and 137.5% for16 d of drought stress, respectively (Fig. 2A-B). However, the JA content was reduced by 76.56% after 16 d of drought stress (Fig. 2C), which demonstrated that drought stress caused water loss accompanied by a series of physiological defense responses in nanmu leaves.

### De novo assembly and annotation of the nanmu transcriptome

Transcriptomic analysis was performed to fully elucidate the transcriptional responses in nanmu to drought stress at 0d-DS and 16d-DS. After obtaining high-throughput reads, 63,380,859 (0d-DS) and 71,899,261 (16d-DS) clean paired-end reads were generated from RNA sequencing and used for de novo assembly (Table S2). A total of 60,250 unigenes were obtained by clustering the assembled transcripts (Table S3). More than 40,000 sequences corresponded to 200–300 bp; this number dramatically decreased when the sequence length was > 300 bp, and the total number gradually decreased as the sequence length increased (Fig. S1a). there were many sequences > 3000 bp accounting for the high abundance (Fig. S1a). The average length of unigenes was 737 bp, and the assembled transcripts were sorted according to length (from long to short). When the length of the accumulated transcript was 50% of the total length, it corresponded to the length of the transcript (N50) of 1878 bp (Table S3). A total of 26,541 unigenes (44.05% of total unigenes) were matched in the five databases. Species distribution analysis showed that 71.38% of these unigenes had top matches in cinnamomum micranthum (Fig. S1b).

**Fig. 3:**
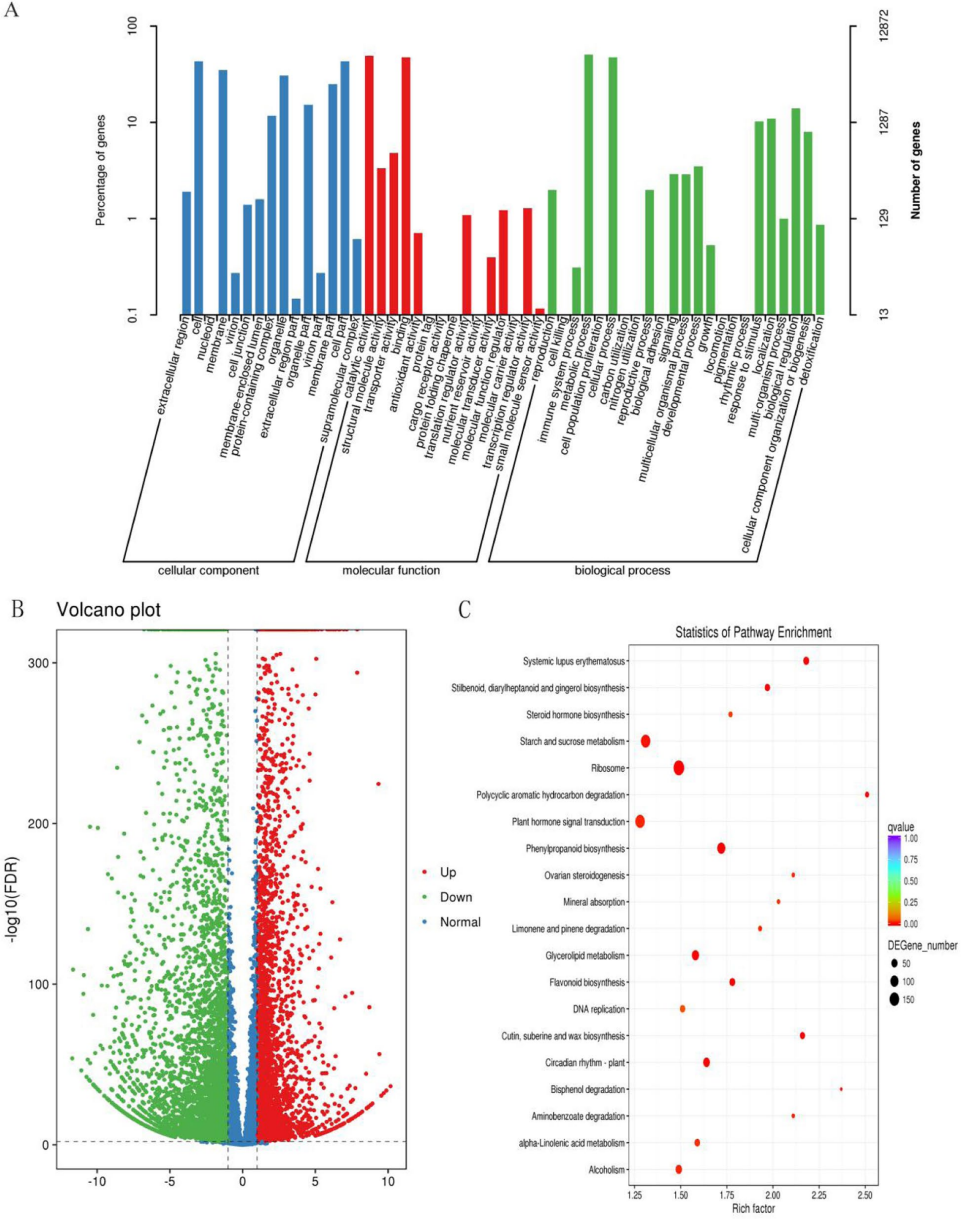
GO classification, Volcano map and. KEGG pathway (**A**) GO secondary classification statistical distribution diagram. Note: ordinate (left), the percentage of the number of genes; ordinate (right), the number of genes aligned. **B** Differentially expressed volcano map. Note: Each point in the differential expression volcano plot represents a gene; log2FC: the logarithm of the fold difference in expression of a gene in two samples; −log10FC(FDR): the negative logarithm of the statistical significance of the change in gene expression. The green dots in the figure represent down-regulated DEGs, the red dots represent up-regulated DEGs, and the blue dots represent non-DEGs. **C** DEGs KEGG pathway enrichment scatterplot. Note: vertical axis: pathway name; horizontal axis: Rich factor (the ratio of the Sample number of DEGs to the background number of DEGs). The size of the dot indicates the number of DEGs in this pathway, and the color of the dot corresponds to different Qvalue ranges. Qvalue is the *P* value after correction for multiple hypothesis testing

Subsequently, functional categorization of the unigenes with Gene Ontology (GO) was performed based on the non-redundant (NR) protein database in NCBI annotation. Unigenes were classified into 53 terms involved in biological processes, cellular components, and molecular functions (Fig. 3A). In the GO categories, “cell” or “membrane part”, “catalytic activity”, and “metabolic process” were dominant among all GO terms. Eukaryotic Orthologous Groups (KOG) analysis using annotated unigenes was further performed to elucidate their putative biological functions. A total of 25 functional classes were matched (Fig. S1c). The “Posttranslational modification, protein turnover, chaperones” [1527 unigenes (10.34%)], “General function prediction only” [3039 (20.58%)], and “Signal transduction mechanisms” [1511 (10.23%)] represented the largest hits (Fig. S1c).

### DEGs enrichment and verification of transcriptome sequencing results

We calculated the expression changes between the control and drought stress in order to identify the DEGs that responded to drought stress. The log2 FC < -1 or > 1, (*P* < 0.01) was defined as significant expression change of control versus drought stress. It was found that 4388 and 3293 unigenes were up and down-regulated, respectively (Fig. 3B). These DEGs were significantly enriched in starch and sucrose metabolism, plant hormone signal transduction, phenylpropanoid biosynthesis, glycerolipid metabolism, flavonoid biosynthesis, alcoholism (Fig. 3C). To verify the results of the RNA-seq data, seven genes related to antioxidant enzymes, aspartate kinase, and aquaporins were randomly detected by Real-time fluorescent quantitative PCR (qRT-PCR). The obtained results were consistent with the up or down-regulation of gene expression in the transcriptomic data (Fig. S2), thus suggesting that the gene expression detected by RNA-seq is reliable.

### Proteomic profiling of nanmu in response to drought stress

**Fig. 4:**
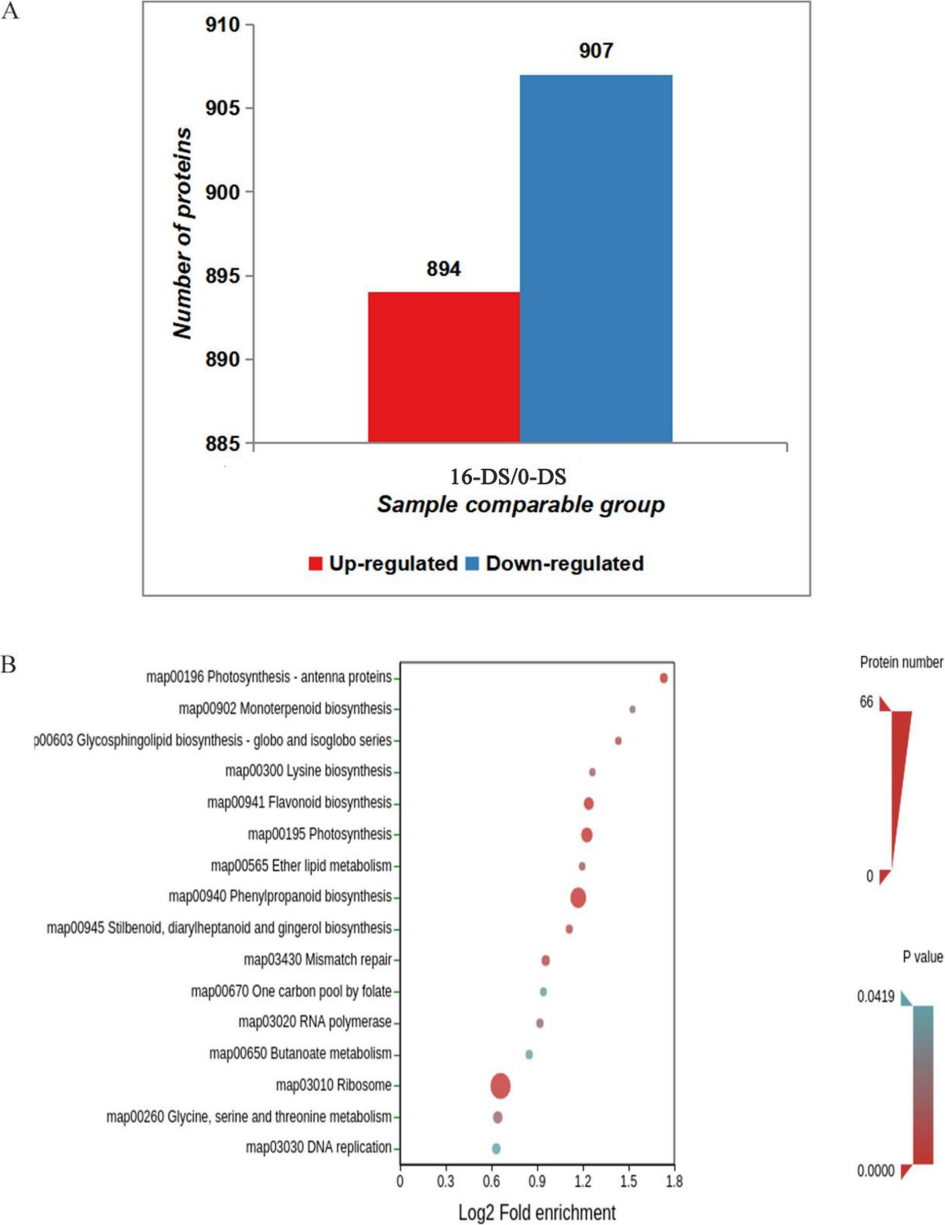
DEPs and KEGG pathway enrichment. **A** Histogram of DEPs. Note:the red represent up-regulated proteins, The green in the figure represent down-regulated proteins. Vertical axis: number of proteins. Horizontal axis: Compared sample name (16-DS/0-DS) when the *P*-value< 0.05, the change of differential expression was more than 1.5 as the threshold of significant up-regulation, and less than 1/1.5 as the threshold of significant down-regulation. **B** DEPs KEGG pathway enrichment. Note: On the vertical axis of the bubble plot is a functional classification or pathway, and on the horizontal axis is a log2 converted value of the ratio of the differential protein in that functional type to the ratio of the identified protein. The color of the circle indicates the enrichment significance *p*-value, and the size of the circle indicates the number of different proteins in functional classes or pathways

To further understand the protein expression changes in nanmu response to drought stress, we performed a 4D label-free proteomic profiling analysis with the same conditions as the transcriptomic analysis. A total of 7051 unique proteins were identified from the 1,287,278 detected peptide spectra (Fig. S3). Expression fold (drought stress to control) was defined > 1.5 or < 0.67 (*p*-value < 0.05) as displaying a significant change. Consequently, we identified 894 up and 907 down-regulated proteins in the nanmu response to drought stress (Fig. 4A). GO, KEGG, and protein domain enrichment analysis identified 1801 DEPs. GO annotation results showed that DEPs were primarily enriched in mRNA modification and flavonol biosynthetic process in the biological processes (Fig. S4a). The DEPs were mainly concentrated in the thylakoid and chloroplast thylakoid, which is consistent with the prediction of subcellular structure and classification statistics (Fig. S4b). With reference to the molecular function, DEPs were primarily related to methyltransferase activity, hydrolase activity, acting on glycosyl bonds and structural constituent of ribosome (Fig. S4c). Kyoto Encyclopedia of Genes and Genomes (KEGG) pathway enrichment showed that many DEPs were significantly enriched in the ribosome-related pathways, such as phenylpropanoid biosynthesis, flavonoid biosynthesis, photosynthesis, and others (Fig. 4B). In addition, protein domain enrichment analysis revealed that peroxidase, glucose-methanol-choline oxidoreductase family, and polyphenol oxidase middle domain-related proteins contained modified protein domains (Fig. S4d).

### Correlation of mRNA and protein profiles

**Fig. 5:**
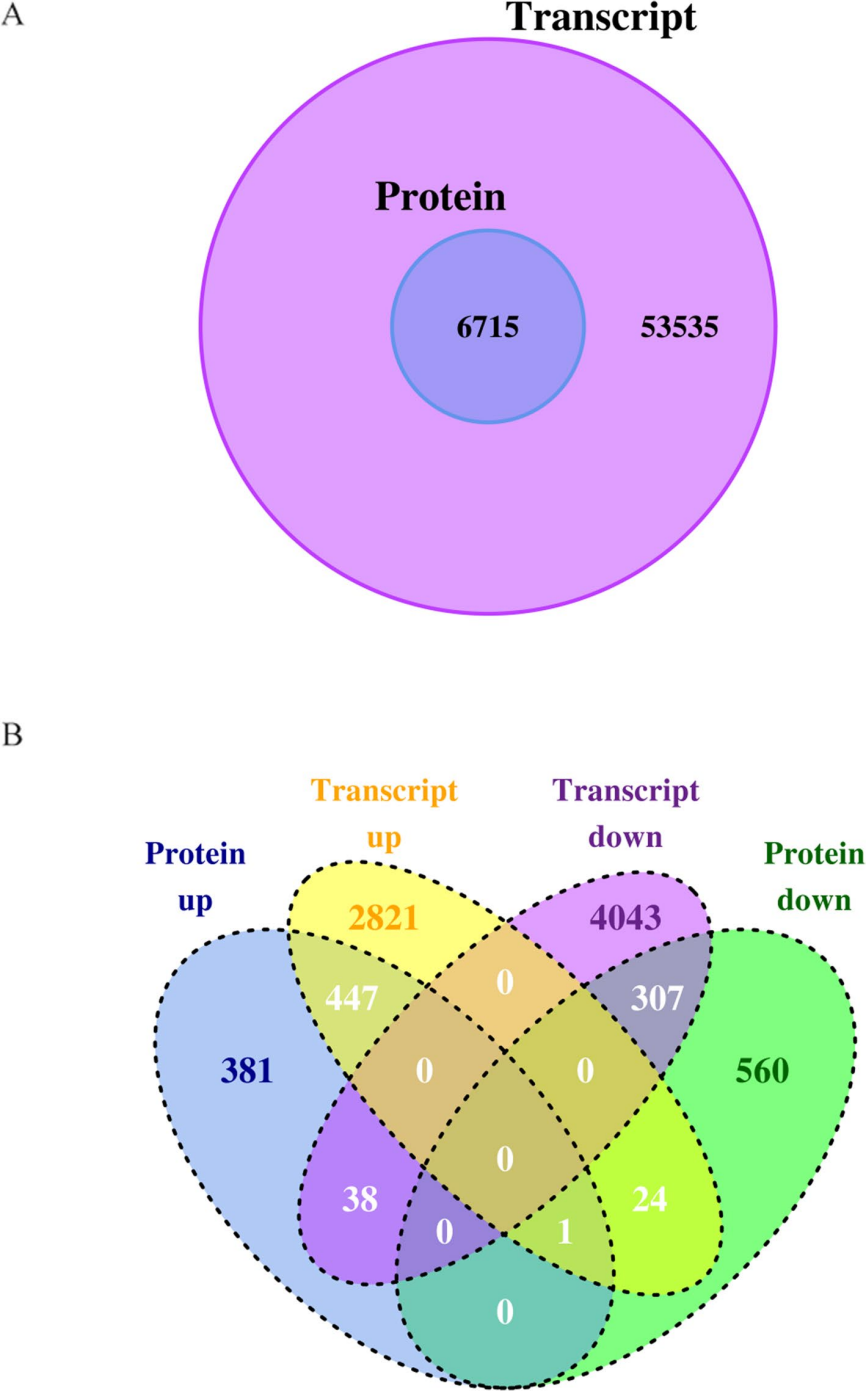
Conjoint analysis Venn diagram. **A** Venn diagram for the quantitative comparison of the transcriptome and proteome. **B** Venn diagram compared between the DEPs and DEGs in the comparison group

We compared the transcriptomic and proteomic data and found that 6715 proteins were quantitatively detected (Fig. 5A). Correlation analysis revealed low correlation coefficients in leaves (*R* = 0.54) (Fig. S5) between the expression levels of all quantified proteins and their corresponding mRNAs. The DEGs and the DEPs with significant changes in expression were further compared. As shown in the Venn diagram (Fig. 5B), transcript up (T^U^) and protein up (P^U^) group had 447 overlaps and transcript down (T^D^) and protein down (P^D^) group had 307 overlaps, which accounted for 54% up and 35% down-regulated proteins, respectively. These results suggested that the transcriptional activity of these genes may modulate their protein levels. In contrast, the 46% up and the 65% down proteins, including the 38 T^D^P^U^ overlaps and 24 T^U^P^D^ overlaps, highlighted a critical role of post-transcription modification in regulating protein expression. It is noteworthy that one gene (TRINITY_DN5471_c0_g1) that was up-regulated corresponded to two proteins (TRINITY_DN5471_c0_g1_m.20867, TRINITY_DN5471_c0_g1_m.20868) that were up and down-regulated, respectively, which may be caused by alternative splicing. Meanwhile, in order to study the effect of post-transcriptional regulation, the GO functional enrichment and KEGG pathway analyses were performed (Fig. S6). The pathways that were primarily enriched included oxidoreductase activity, metabolic pathways metabolism, phenylpropanoid biosynthesis, hormone biosynthesis, and photosynthesis.

### The critical DEGs and DEPs related to nanmu response to drought stress

**Fig. 6:**
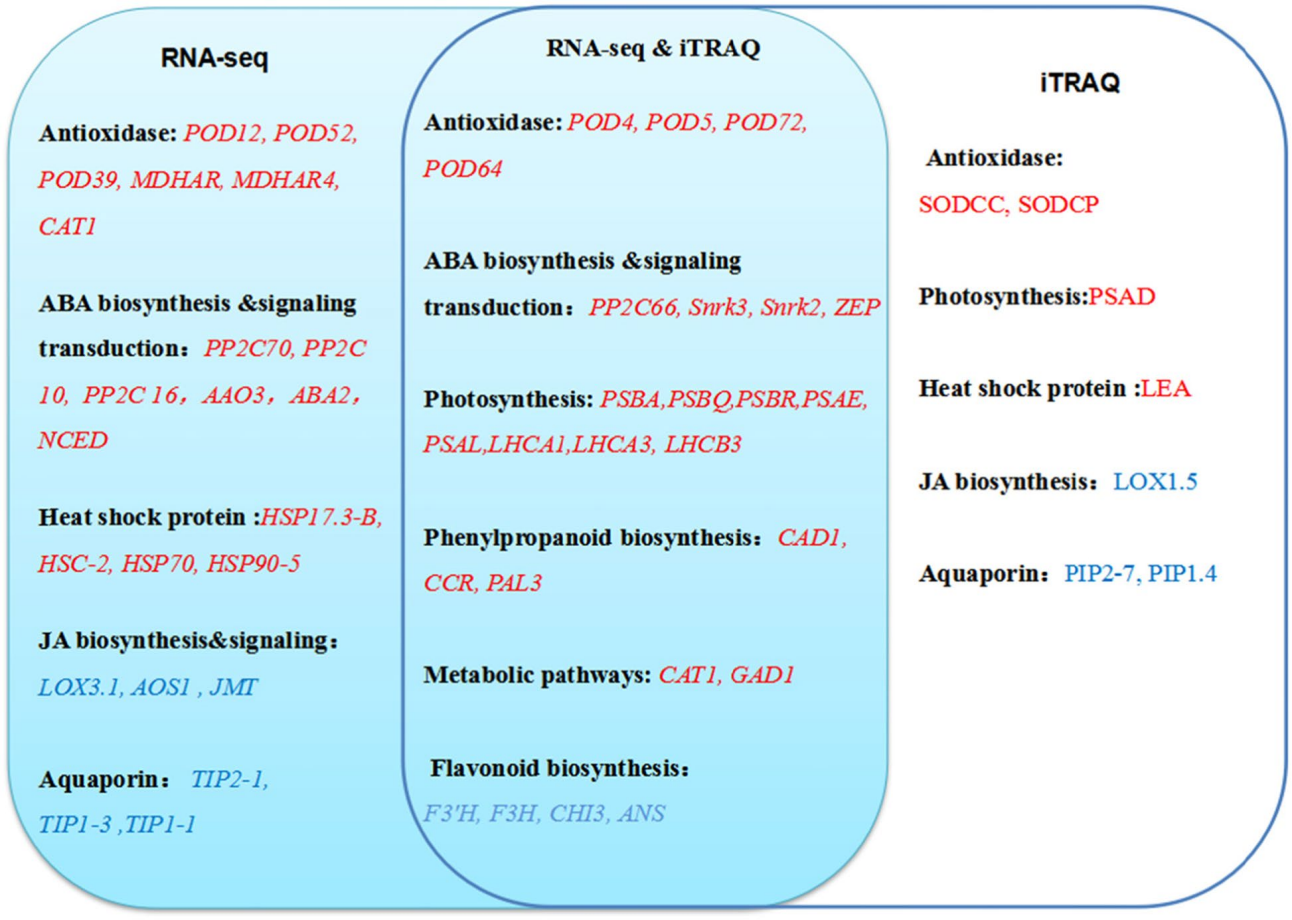
Pathway analysis of nanmu affected by drought stress using RNA-seq and iTRAQ. Note: Refer to Ding et al. [25]

Based on the integrative proteomic and transcriptomic analyses, we focused on critical proteins and genes implicated in several aspects of physiological and biochemical responses to drought stress. These up-regulated genes/proteins were enriched in phenylpropanoid biosynthesis, metabolic pathways, and photosynthesis (Fig. S6). Consistently, cinnamoyl-CoA reductase 1(*CCR1*), phenylalanine ammonia-lyase 3 (*PAL3*), and cinnamyl alcohol dehydrogenase 1(*CAD1*) were involved in the phenylpropanoid biosynthesis (Table S4 and Fig. 6). Catalase isozyme 1 (*CAT1*) and glutamate decarboxylase 1 (*GAD1*) were involved in the metabolic pathways (Table S4). Drought significantly induced proteins involved in photosynthesis and photosynthesis-antenna proteins, including Photosystem II (PSBA, PSBQ), Photosystem I (PSAE, PSAL). Light-harvesting chlorophyll protein (LHCA1, LHCA3, LHCB3) was significantly up-regulated at the transcript level and protein level. Besides three genes, peroxidase (*POD4, POD5, POD64,* and *POD72*) that were involved in ROS scavenging were also significantly up-regulated (Table S4). Drought also greatly induced the expression of genes associated with ABA biosynthesis and Signal transduction, including protein phosphatase 2C (*PP2C66*), Serine/threonine-protein kinase (Snrk3 and Snrk2), and zeaxanthin epoxidase (*ZEP*). These down-regulated genes were enriched in flavonoid biosynthesis (Fig. 6). chalcone--flavonone isomerase 3 (*CHI3*), flavanone 3-dioxygenase (*F3H*), flavonoid 3’monooxygenase (*F3’H*), and leucoanthocyanidin dioxygenase (*ANS*) were found to be involved in flavonoid biosynthesis (Table S4).

Next, we focused on the genes/proteins that only changed at the transcriptomic or proteomic level in specific pathways. There were 2841 up-regulated and 4043 down-regulated DEGs whose corresponding proteins were not differentially expressed (Fig. 5). Drought also significantly induced the expression of abiotic stress genes. Phosphatase 2C (*PP2C 10,PP2C70,* and *PP2C 16*), Indole-3-acetaldehyde oxidase (*AAO3*), Secoisolariciresinol dehydrogenase (*ABA2*), carotenoid cleavage dioxygenase (*NCED*) were involved in the ABA biosynthesis and Signal transduction (Fig. 6). Furthermore, most of these genes were associated with HSPs (*HSP17.3-B, HSC-2, HSP70,* and *HSP90–5*) (Table S4 and Fig. 6).

There were 381 up-regulated and 560 down-regulated DEPs whose corresponding genes were not differentially expressed (Fig. 5). As expected, drought significantly induced proteins involved in photosynthesis, including Photosystem I reaction center subunit II (PSAD). Late embryogenesis abundant protein Lea14-A (LEA) protein was significantly up-regulated at the protein level (Table S4). Drought also significantly and greatly induced the proteins involved in redox-related Superoxide dismutase (SODCC, SODCP) response to oxidative stress. On the contrary, it dramatically suppressed the expression of Glutathione S-transferase (GSTF9, GSTF17) (Table S4).

## Discussion

### Physiological responses to drought stress in nanmu

Drought is considered to be the main abiotic environmental stress that is not conducive to plant survival and growth [29]. Plant adaptation to drought stress involves a series of physiological changes [30, 31]. RWC is generally considered as an indicator of plant cells’ water status under drought stress, whose variation reflects the drought resistance of the plant [32, 33]. Under abiotic stress conditions, excessive free radicals are formed in plants, adversely affecting plant growth and development. ROS, such as H_2_O_2_ and O2·−, are some of the most damaging molecules in plants [34]. In this work, the significant increase observed in H_2_O_2_ and O2· − levels under drought stress was consistent with the results of previous studies, thus indicating that ROS scavenging may be related to the drought response of nanmu. At the same time, under adverse conditions, antioxidant enzymes (CAT, SOD, and POD) could scavenge excess free radicals, which prevents free radicals from damaging the plant cell membrane system and other macromolecules and increases the plant’s resistance to stress via antioxidant enzyme system activation [35]. Under drought conditions, plants accumulate osmotic adjustment substances such as PRO and soluble sugars to maintain cell turgor, cell membrane stability, and protein function and promote photosynthesis [36]. In the present work, the SS and Pro contents followed similar trends during the drought period, revealing a significant increase. These results were consistent with previous research [37], indicating that SS and Pro have a key role in drought tolerance.

In this study, physiological data were investigated in nanmu leaves at five-time points (within 16 days) under drought stress. This study is similar to previous studies on the physiological, osmotic regulation, and antioxidant defense systems of nanmu [28]. The physiological and biochemical indicators of these studies (including PRO content and MDHAR, GST, and POD activity) revealed the induction of response to drought stress in nanmu. During drought stress, *P. communis* and *P. scoparia* showed both high APX and MDHAR activities [38]. In this work, the MDHAR activity value significantly increased in the middle of the drought and remained stable in the later period (Fig. 1E), which indicated that MDHAR is likely involved in the drought response in nanmu. GSTs are multifunctional enzymes that have a crucial role in cellular detoxification and oxidative stress tolerance [39]. In the present study, GST activity first increased and then decreased, which indicated that GST participated in the response under drought conditions. POD is one of the most important enzymes that further convert H_2_O_2_ into H_2_O and O2. Also, the damage caused by ROS is eliminated from plants [40]. In this study, POD activity increased in the pre-drought period, which might be a form of adaptation for alleviating oxidative stress. Subsequently, POD activity sharply decreased. These results indicated that POD is sensitive to abiotic stress and may be important for the drought tolerance mechanism of nanmu seedlings.

### Chlorophyll metabolism and photosynthesis

Drought stress can lead to plant dehydration and inhibit plant growth by hindering the physiological and metabolic processes of nanmu leaves. Magnesium-chelatase subunit (ChlH, ChlD) protein was found to be down-regulated under drought stress in the present study (Table S4). It is one subunit of the magnesium-chelatase complex, which has an essential role in chlorophyll biosynthesis. One of the inhibitory effects of drought on plant photosynthesis is decreased chlorophyll content in leaves [41]. In this study, Chl a and b of nanmu were significantly reduced after the 16d-DS treatment, which may be attributed to the down-regulation of ChlH and ChlD (Fig. 1C). The activity of Pheophorbide a oxygenase (PAO), a key chlorophyll catabolic enzyme, was enhanced by drought stress (Table S4).

In addition, photosynthesis and photosynthesis-antenna proteins were both up-regulated (Table S4), whereas one gene involved in the photosynthetic electron transport, Ferredoxin-3(PETF), was down-regulated (Table S4). The results showed that drought stress induced the expression of genes and proteins, including PSII and PSI, which was conducive to stabilize the structure of PS II and PSI from being damaged by drought stress, repair the damaged PSI and PSII, and stabilize the redox state of photosynthetic electron transport chain. However, the significant down-regulation of PETF gene inhibited the activity of photosynthetic electron transport chain. Drought stress not only affected the light reaction process, but also affected the dark reaction process. This study found that the down-regulation of glyceraldehyde 3-phosphate dehydrogenase (GAPDH) in Kelvin pathway may lead to the decrease of photosynthetic carbon assimilation efficiency (Table S4), and then affect the photosynthetic activity of nanmu. Drought stress induced up-regulated expression of light response related proteins, which may be a mechanism of light protection and light adaptation of Phoebe to drought adverse environment.

### Roles of flavonoid and phenylpropanoid biosynthesis genes in drought

**Fig. 7:**
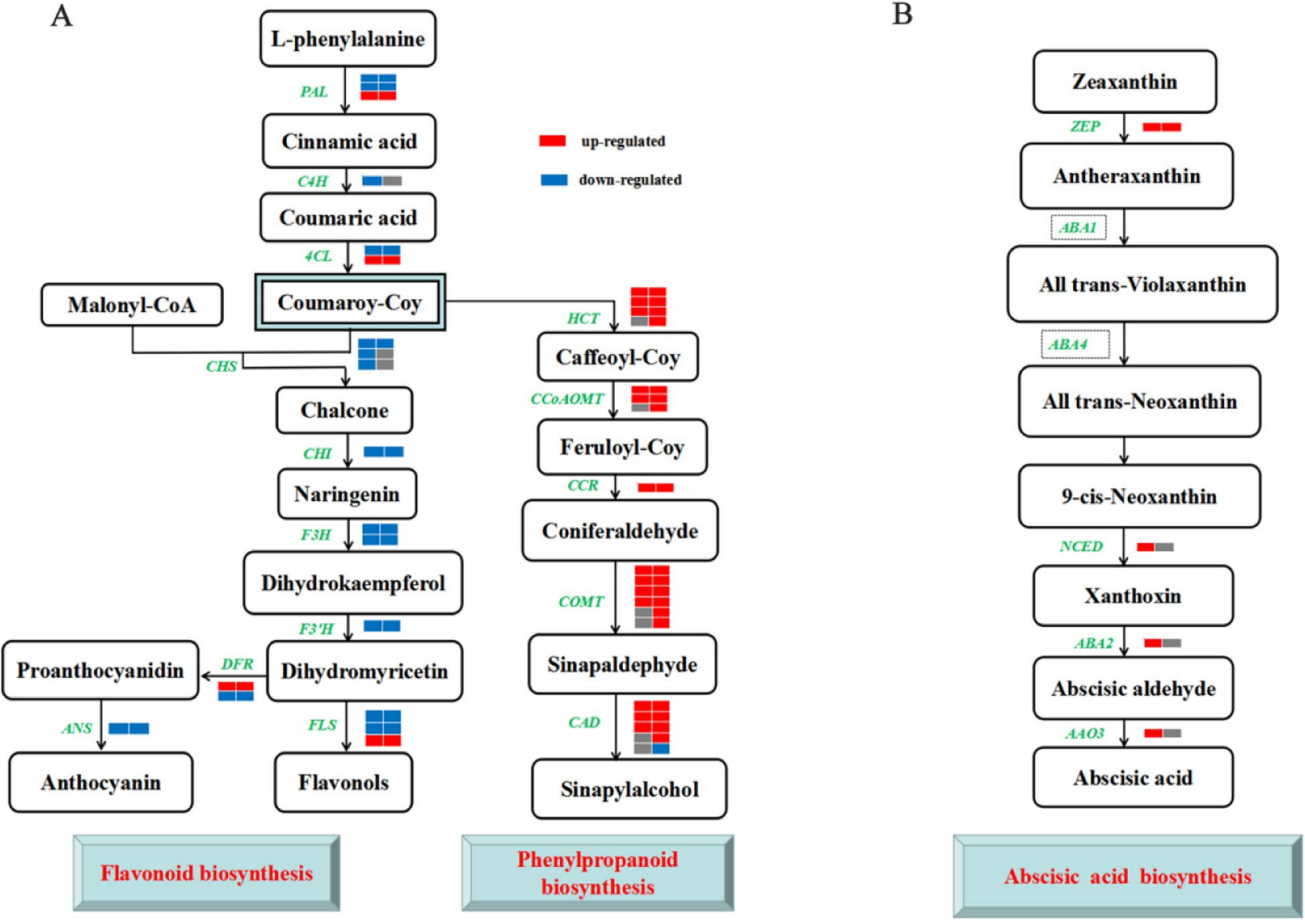
Heatmap of DEGs and DEPs related to biosynthesis. **A** flavonoid biosynthesis and phenylpropanoid biosynthesis. **B** Abscisic acid biosynthesis. Up- and down-regulated DEGs/DEPs were indicated by red and blue colors, respectively. Nonsignificant genes/proteins were indicated by a gray color

Secondary metabolites such as phenylpropanoids, anthocyanins, and flavonoids are important compounds essential for plant acclimation [42]. Water deficit can significantly affect the expression of genes related to phenylpropanoid and flavonoid pathways [17]. The phenylpropanoid pathway serves as a source of metabolites in plants, as well as a starting point for the lignans and flavonoids [42]. In the present study, 13 and 25 genes were identified for flavonoid and phenylpropanoid biosynthesis pathways, respectively (Fig. 7A). Most genes in the flavonoid pathway was down-regulated and phenylpropanoid pathway was significantly up-regulated at the mRNA and protein levels, respectively. thus indicating that these genes were mainly affected by transcriptional regulation (Table S4). These results also suggested that integrative transcriptomic and proteomic analysis might be important for the investigation of the expression of flavonoid and phenylpropanoid biosynthesis genes, at least under the condition of water deficit in nanmu.

### The role of hormone biosynthesis in drought

ABA, JA, and SA are considered important signals in abiotic stress response and drought stress memory [43, 44]. As far as an abiotic stress response to plant hormones, high ABA content is an important indicator of drought, high temperature, high salt, and cold [45, 46]. In this study, the ABA content in nanmu leaves showed a sharp upward trend with the continuous drought stress (Fig. 2A). The increased ABA content in nanmu leaves facilities stomatal movement to reduce transpiration water loss and maintain cell viability [47], which is consistent with the previously published results [48]. Plants synthesize abscisic acid biosynthesis (ABA) using the carotenoid pathway. Zeaxanthin epoxidase (*ZEP*) catalyzes the conversion of zeaxanthin to all-trans-violaxanthin via antheraxanthin [49], after which 9′-cis-neoxanthin can be oxidatively cleaved by the 9-cis-epoxycarotenoid dioxygenase (*NCED*) [50]. Genetic and biochemical studies revealed that the cleavage of 9-cis-xanthophylls produces a C_15_-apocarotenoid and xanthoxin [51]. A short-chain alcohol dehydrogenase encoded by AtABA2/AtGIN1 then converts xanthoxin into abscisic aldehyde, which is eventually oxidized to ABA by AtABA3, an abscisic aldehyde oxidase (*AAO3*) [52] (Fig. 7B). These data revealed an intact biosynthesis pathway of ABA. In the present study, the identified synthesis-related genes, including *ZEP*, *NCED*, *ABA2*, and* AAO3* in ABA synthesis, the gene expression levels of *ZEP*, *NCED*, *ABA2*, and *AAO3* were significantly up-regulated in nanmu (Table S4). The up-regulated genes in this study were consistent with the results of X U et al. [53], which suggested they acted as important factors in core ABA biosynthesis (Fig. 7B) Normally, ABA is involved in drought signal transduction in plants, and the expression of many genes is altered (repressed or induced) under drought conditions. In this work, the gene expression levels of Serine/threonine-protein kinase (Snrk2, Snrk3), abscisic acid receptor PYL6 (PYL), and protein phosphatase 2C (PP2C) were significantly up-regulated in nanmu (Table S4). PP2Cs have an important role in core ABA signaling pathways [54]. PYLs have differing binding properties with ABA, and selectively interact with PP2Cs [55]. In the absence of ABA, PP2Cs interact with and repress SnRK2s to block ABA signaling [56]. Interestingly, the expression changes of these ABA signaling genes were consistent with the increase in ABA levels, strongly supporting their involvement in ABA-dependent drought response in nanmu. Moreover, aquaporins contribute to ABA-triggered stomatal closure through OST1-mediated phosphorylation [57]. Arabidopsis *AtPIP2;1* and AtPIP2;2 are down-regulated under drought stress [58]; however, AtPIP1;3, AtPIP1;4, and few other AtPIPs are up-regulated [59]. Grondin et al. found that the expression of PIP2;1, PIP1;3, PIP2;2, PIP1;1, PIP1;2, and PIP2;8 were significantly reduced in the roots of six rice varieties under drought stress. In this study, the majority of the PIP genes were down-regulated under drought stress (Fig. 6 and Table S4), which is consistent with the results reported by Grondin et al. in their study [57].

JA is essential to signal transducers that drive the expression of plant defense genes in response to environmental stress [60]. JA is extensively found in higher plants and can significantly enhance plant resistance to insects, diseases, and drought. Dong et al. [61] found that external application of methyl jasmonate (MeJA) moderately reduced the damage to rice seedlings caused by drought stress. Xu and colleagues [53] have pointed out that JA immediately accumulates when millet is under drought stress, and the cell turgor pressure decreases; however, JA cannot regulate water stress for an extended period of time [53], which is different from the adversity response hormone ABA. Dhakarey et al. [62] used JA to synthesize the CPM2-deficient mutant in rice and found that under drought stress, the mutant had higher efficiency of water use, stronger metabolism, and significantly increased secondary metabolites, thus suggesting that endogenous JA was a negative regulator under drought stress. In the present study, the JA content in nanmu leaves decreased after the 16-DS treatment. Therefore, based on the current experiments, it was not possible to determine whether there is a transient accumulation effect of JA, so further continuous measurement experiments are required. Yet, the identified synthesis-related genes, including Allene oxide synthase 1(AOS1) and linoleate 13S-lipoxygenase 3–1 (LOX3.1) in JA synthesis, were up-regulated in transgenic levels (Fig. 6 and Table S4).

Compared with these two hormones, the SA effect under drought stress has been rarely reported. Our results revealed that SA content in nanmu leaves increased after the 16-day drought treatment. This was related to the up-regulation of the key enzymes responsible for the SA biosynthesis, including mitogen-activated protein kinase 4 (*MPK4*)*,* caffeic acid 3-O-methyltransferase 1 (*OMT1*)*,* caffeic acid 3-O-methyltransferase (*COMT1*)*,* transcription factor TGA4 (*TGA4*)*,* and caffeic acid 3-O-methyltransferase 1 (*HOMT1)* (Table S4). Further investigation is needed to determine whether the SA response is related to drought resistance.

### Roles of HSPs in drought stress

HSPs have an important role not only in heat stress but also under other stressful conditions, including drought, salt, oxidative stress, and cold [63]. These proteins have essentially been implicated in the innate immunity and abiotic stress tolerance in higher plants. HSPs usually act as molecular chaperones to protect cells against multiple stresses through signaling. At the same time, HSPs can also function via protein folding [63]. In the present study, it was clearly observed that the majority of HSPs, such as HSP17.3-B, HSC-2, HSP70, and HSP90–5, whose corresponding proteins were not differentially expressed in nanmu, were dramatically up-regulated (Table S4).

It has been suggested that HSPs are involved in drought stress through transcriptional regulation. Hsp70 is involved in stomatal closure and regulation, transcriptional and physiological regulation in response to ABA signaling [64]. HSPs can also participate in ROS scavenging genes to protect plants from drought stress. Furthermore, late embryogenesis abundant protein Lea14-A (LEA) protein was significantly up regulated at the protein level (Table S4). Some scholars have shown that the expression of LEA is related to the enhanced tolerance of plants to abiotic stress [65]. Xiao et al. [66] found that overexpression of LEA-related genes in rice could significantly improve plant drought resistance, suggesting that these proteins may protect biological macromolecules and prevent water loss in plant cells.

## Conclusion

In this study, physiological and biochemical indices were analyzed, and transcriptomic and proteomic analyses were performed on nanmu seedlings treated with natural drought stress. We found that nanmu seedlings could regulate the antioxidant system, osmotic regulation, hormone levels, chlorophyll metabolism, photosynthesis pathway, and photosynthesis-antenna proteins, thus alleviating the damage caused by drought stress. Furthermore, we screened and identified the key metabolic pathways, genes, and proteins of nanmu that alleviate drought stress. Our results revealed that drought stress could induce nanmu oxidoreductase activity, metabolic pathways metabolism, phenylpropanoid biosynthesis, hormone biosynthesis, and expression of genes and proteins related to photosynthesis, effectively promoting the synthesis and metabolism pathways of key metabolites. This improved osmotic regulation capacity, antioxidant capacity, and water retention capacity of cells. In conclusion, we conducted an in-depth study on the physiological and biochemical processes and nanmu’s transcriptional and protein expression levels in response to drought stress. The reported findings lay a foundation for the physiological and molecular mechanism of drought resistance of *P. zhennan,* thus providing a reference for further in-depth analysis of the molecular mechanism of drought resistance.

## Methods

### Plant materials and drought stress treatments

The experiment was conducted at the Key Laboratory of Plant Resources Conservation and Germplasm Innovation in the mountainous region (Ministry of Education), Guizhou University, China. Healthy two-year-old nanmu plants with consistent growth were collected from Shiqian County (Guizhou Province). Two-year-old nanmu was obtained with permission from Shiqian County Changrong Forestry Office and transplanted into plastic pots (24 × 19 × 5 cm) filled with nutrient soil (pH 6.0, 15% organic matter, and 20% humic acid). The plants were further grown under a 12 h light (24 °C)/12 h dark (20 °C) photoperiod with 8000Lx-10,000 Lx photosm^− 2^·s^− 2^ light intensity and 60% relative humidity in a growth chamber (Shenzhen Samkoon Technology Corporation Ltd., China) for 16 days.

### Physiological and biochemical experiments

The leaves of nanmu seedings (three biological replicates) were collected for drought stress at 0, 4, 8, 12, and 16 days. The RWC of leaves was calculated using the formula previously described by Ge et al. [67]. PRO, Chl a and b Chl b, POD, MDHAR, and GST were each assayed with PRO, Chl a/b, POD, MDHAR, H2O2, SS, O2· − and GST assay kits, respectively (Suzhou Comin Biotechnology Co. Ltd., China). ABA, SA, and JA content were measured with ultra-performance liquid chromatography-tandem MS (UPLC-MS/MS). The least significant difference (LSD) method was used to test the significance of the mean of the three replicates.

### Construction of transcriptome sequencing library

The RNA-seq library preparation and sequencing were conducted by the Biomics Corporation (Beijing, China). Briefly, plants grown for 0 and 16 d (drought-stressed) were selected for the assay. RNA purity was verified using the NanoPhotometer® spectrophotometer (IMPLEN, CA, USA). RNA concentration was measured with the Qubit® RNA Assay Kit using the Qubit® 2.0 Fluorometer (Life Technologies, CA, USA). After testing, the concentration and purity of the RNA were evaluated using the RNA Nano 6000 Assay Kit on the Agilent Bioanalyzer to evaluate the RNA integrity using the 2100 system (Agilent Technologies, California, USA). The library was constructed after high-quality RNA was obtained, and the qualified library was sequenced using the Illumina (New England Biolabs, USA) sequencing platform. Trinity [68] software was used to assemble the sequences after high-quality clean data were selected. The number of the read counts compared to each gene for each sample were expressed as (Fragments Per Kilobase of transcript per Million mapped reads) FPKM. The *P*-value was adjusted using the Benjamini and Hochberg method to control error discovery. Genes with adjusted *P*-values < 0.01 identified by the DESeq2 software [69] were designated as differentially expressed. Gene functions were annotated based on the NCBI non-redundant protein sequences (Nr) and Clusters of Orthologous Groups of proteins (KOG/COG). Gene Ontology (GO) enrichment analysis of the DEGs was implemented with the GOseq R packages [70], which can adjust for gene length bias in DEGs. Kyoto Encyclopedia of Genes and Genomes (KEGG) [71] is a large-scale molecular database generated via genome sequencing and other high-throughput experimental technologies (http://www.genome.jp/kegg/). KOBAS [72] software was used to test the statistical enrichment of DEGs in the KEGG pathways.

### Protein extraction and trypsin digestion

Plant total protein extraction was conducted according to Zhou et al. [73] method. Two hundred mg of fresh sample were weighed into a pre-cooled mortar and ground with liquid nitrogen. According to Zhao et al. [74] method, a four-fold volume of phenol extraction buffer was added for ultrasonic cracking and an equal volume of tris balanced phenol (Solarbio, China) was added for centrifugation. The supernatant was removed into a centrifuge tube for later use, and the protein concentration was determined using the BCA protein kit (Biyuntian, China). Trypsin digestion protocol was done as follows: first, the extracted proteins were reduced with 5 mM dithiothreitol at 56 °C for 30 min. Next, each sample was alkylated with 11 mM iodoacetamide and incubated for 15 min at room temperature in the dark. Subsequently, the sample was diluted by adding 100 mM tetraethylammonium bromide (TEAB) to a urea concentration < 2 M. Finally, 2% trypsin was added for overnight digestion.

### LC-MS/MS and database analyses

The peptides were dissolved in 0.1% formic acid (solvent A) and loaded onto the reversed-phase analytical column. The gradient was comprised of an increase from 6 to 24% of solvent B (0.1% formic acid in acetonitrile) over 70 min, 24 to 35% in 14 min, increasing to 80% in 3 min, and then holding at 80% for the last 3 min. The flow rate was maintained at 450 nL/min. The peptides were subjected to a capillary source followed by tandem mass spectrometry (MS/MS) in tims-TOF Pro (Bruker, Germany) coupled online to the UPLC. The ion source voltage was set to 2.0 kV. The resulting MS/MS data were processed using the Maxquant search engine (v.1.6.6.0) [75]. Moreover, Correlation coefficients between the expression levels of DEPs and their corresponding mRNAs were calculated by the Pearson correlation test.

### Quantitative real-time PCR

Reverse transcription of RNA was performed using the PrimeScript™ RT reagent Kit with gDNA Eraser (Perfect Real Time) (TaKaRa, Japan). The expression level of seven genes (Table S1) was verified by real-time PCR analysis using Luna® Universal qPCR Master Mix (New England Biolabs, USA) on the CFX Connect™ (BIO-RAD, USA) system. The thermocycler was set to the following program: 95 °C for 3 min; 95 °C for 10 s, 40 cycles; and 55 °C for 20 s. A melting curve was generated by heating the sample to 95.0 °C. The comparative Ct method was used to analyze the real-time PCR data [76].

## Supplementary Information

**Additional file 1: Figure S1.** Sequence length distribution diagram and classification diagram. (a) Sequence length distribution diagram of assembly results. (b) NR annotated species classification map. (c) KOG annotation classification **Figure S2.** Real-time quantitative PCR analysis of drought stress. **Figure S3.** Basic statistical graph of mass spectra data results. **Figure S4.** Bubble charts provide the results for the top 20 categories of most significant enrichments. (a) Biological Process. (b) Cellular Component. (c) Molecular Function. (d) Protein domain enrichment. **Figure S5.** A scatter plot of the transcript and its corresponding protein expression. **Figure S6.** KEGG pathway results. **Figure S7.** Heatmap of DEGs and DEPs related to HSPs in Nanmu. **Table S1.** A detailed list of primer sequences used in this study. **Table S2.** The statistical results compared with the reference sequence. **Table S3.** Statistical table of assembly results. **Table S4.** Overlap of DEGs and DEPs in nanmu (genotype).

## Data Availability

The data sets generated and analyzed in our study are available in the NCBI Sequence Read Archive under the accession number of RJNA778346.

## Abbreviations

COG/KOG: Clusters of Orthologous Groups of proteins
KEGG: Kyoto Encyclopedia of Genes and Genomes
GO: Gene Ontology
DEGs: Differentially expressed genes
DEPs: Differentially expressed proteins
ET: Ethylene
SOD: Superoxide dismutase
CAT: Catalase
RWC: Leaf relative water content
ROS: Reactive oxygen species
PRO: Proline
MDHAR: Monodehydroascorbate reductase
GST: Glutathione S-transferase
Chl: Chlorophyll
Chl a: Chlorophyll a
Chl b: Chlorophyll b
ABA: Abscisic acid
JA: Jasmonic acid
SA: Salicylic acid
MDA: Malondialdehyde
BRs: Brassinosteroids
0d-DS: 0-day Drought-stressed
16d-DS: 16-day Drought-stressed
O2· −: Superoxide anion
H_2_O_2_: Hydrogen peroxide
SS: Soluble sugar
T^U^: Transcript up
P^U^: Protein up
T^D^: Transcript down
P^D^: Protein down
