# Supplementary material for: Transcriptomic and proteomic analyses uncover the drought adaption landscape of Phoebe zhennan
Source: BMC Plant Biol. 2022 Mar 3;22:95. doi: 10.1186/s12870-022-03474-3 (PMC8892755; doi:10.1186/s12870-022-03474-3)
Supplement: Supplementary file 1 — Additional file 1: Figure S1. Sequence length distribution diagram and classification diagram. (a) Sequence length distribution diagram of assembly results. (b) NR annotated species classification map. (c) KOG annotation classification Figure S2. Real-time quantitative PCR analysis of drought stress. Figure S3. Basic statistical graph of mass spectra data results. Figure S4. Bubble charts provide the results for the top 20 categories of most significant enrichments. (a) Biological Process. (b) Cellular Component. (c) Molecular Function. (d) Protein domain enrichment. Figure S5. A scatter plot of the transcript and its corresponding protein expression. Figure S6. KEGG pathway results. Figure S7. Heatmap of DEGs and DEPs related to HSPs in Nanmu. Table S1. A detailed list of primer sequences used in this study. Table S2. The statistical results compared with the reference sequence. Table S3. Statistical table of assembly results. Table S4. Overlap of DEGs and DEPs in nanmu (genotype). [file 12870_2022_3474_MOESM1_ESM.docx]

**Additional file 1**


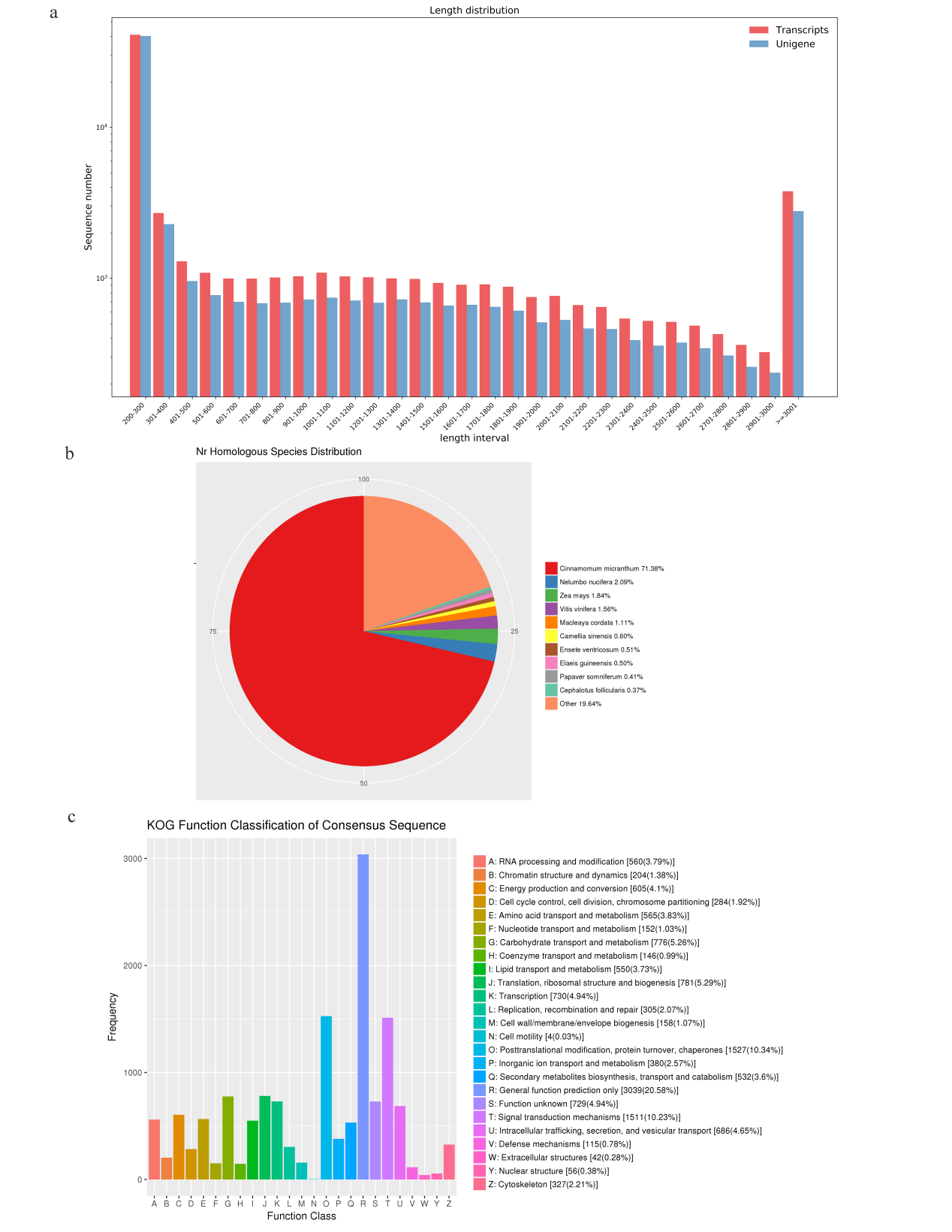


**Figure S1.** Sequence length distribution diagram and classification diagram. (a) Sequence length distribution diagram of assembly results. (b)NR annotated species classification map.(c) KOG annotation classification,Note: The horizontal axis is the content of each KOG classification; the vertical axis is the number of genes.


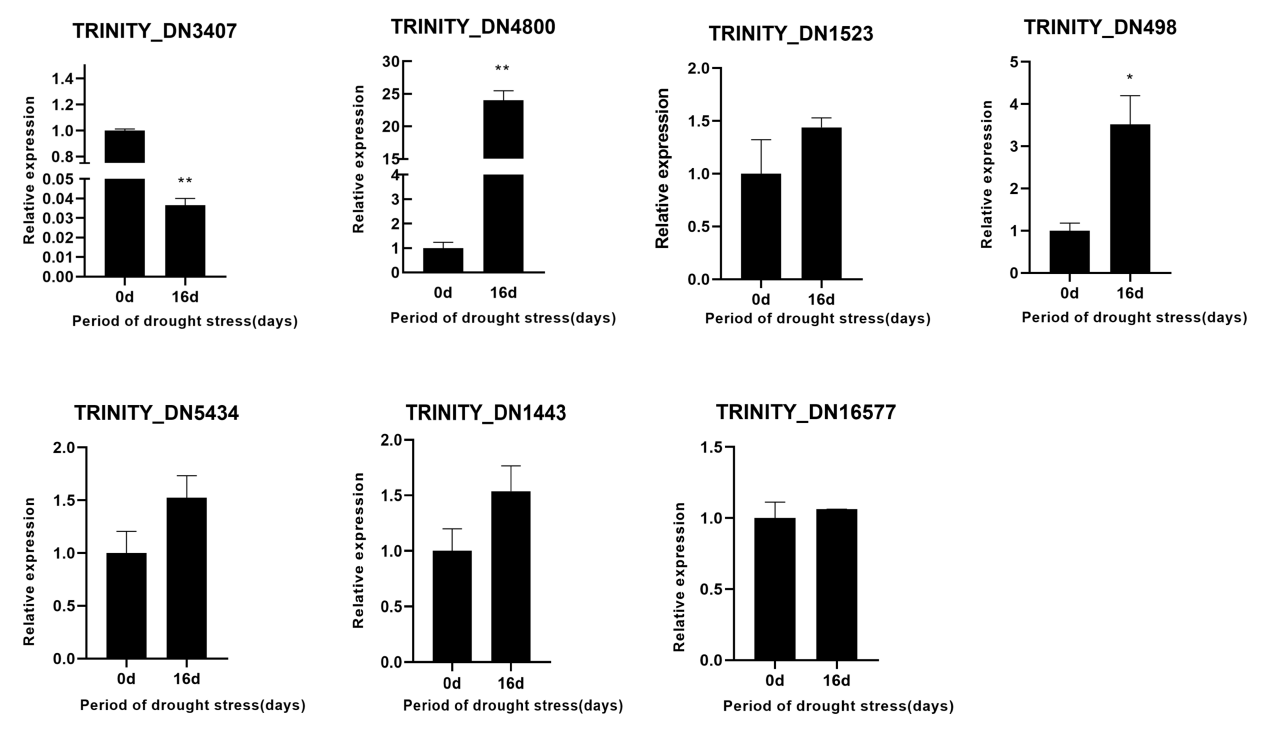


**Figure S2.** Real-time quantitative PCR analysis of drought stress.Data are means of three biological replicates and error bars are + SE from three independent experiment.Asterisks indicate signiflcant differences by Tukey LSD test(*P<0.05).


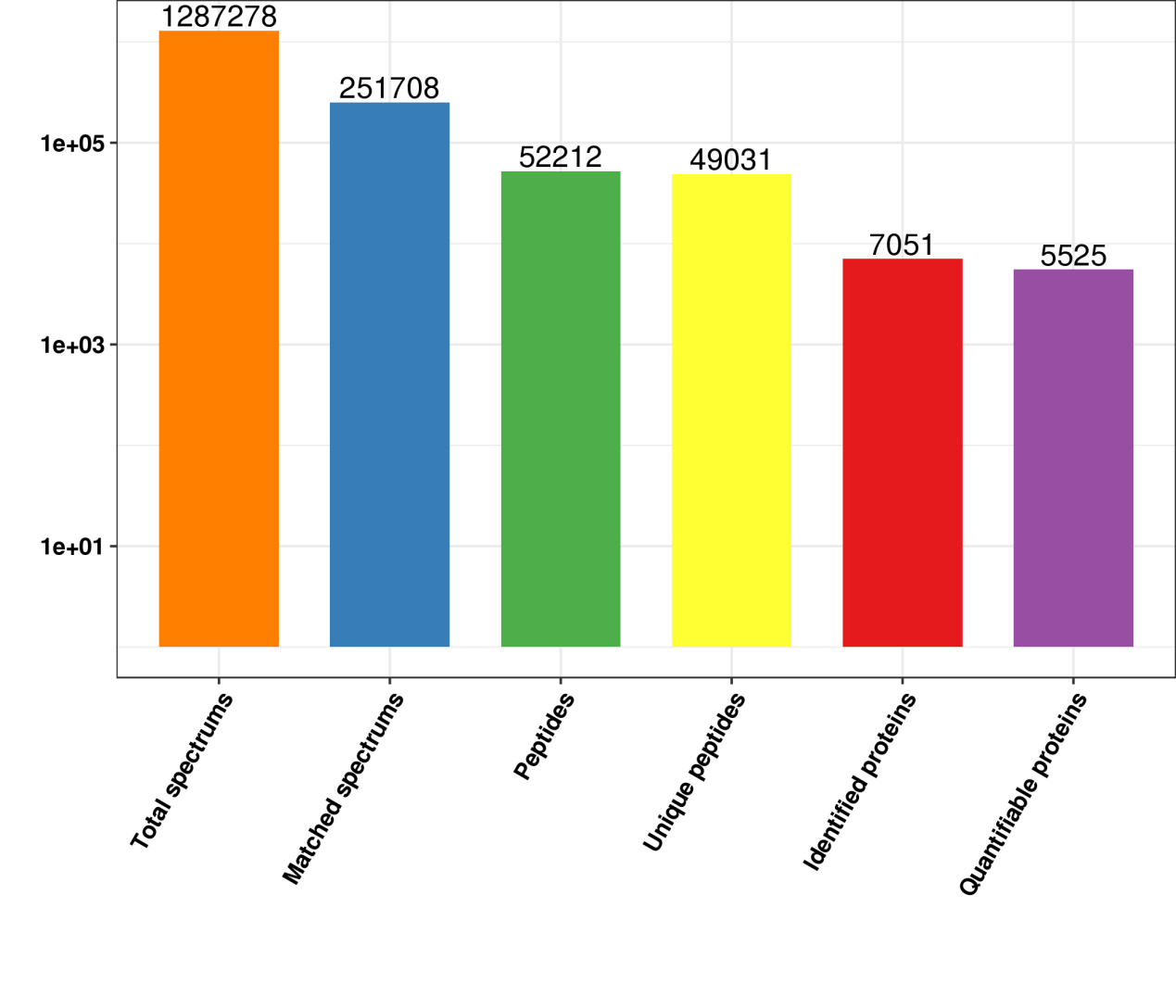


**Figure S3.** Basic statistical graph of mass spectra data results


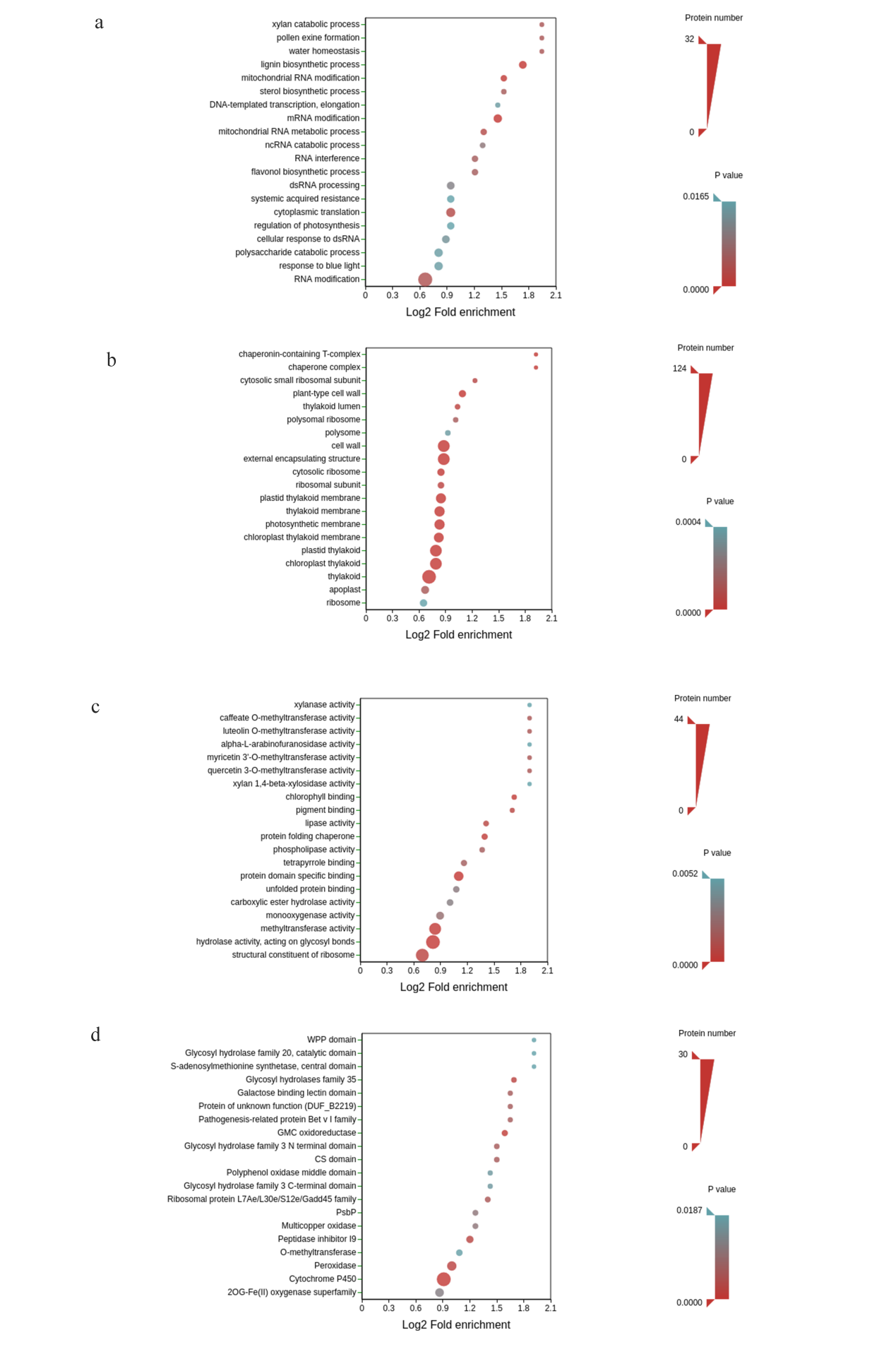


**Figure S4** Bubble charts provide the results for the top 20 categories of most significant enrichments. (a)Biological Process.(b)Cellular Component.(c) Molecular Function. (d) Protein domain enrichment. Note: On the vertical axis of the bubble plot is a functional classification or pathway, and on the horizontal axis is a log2 converted value of the ratio of the differential protein in that functional type to the ratio of the identified protein.The color of the circle indicates the enrichment significance p-value, and the size of the circle indicates the number of different proteins in functional classes or pathways.


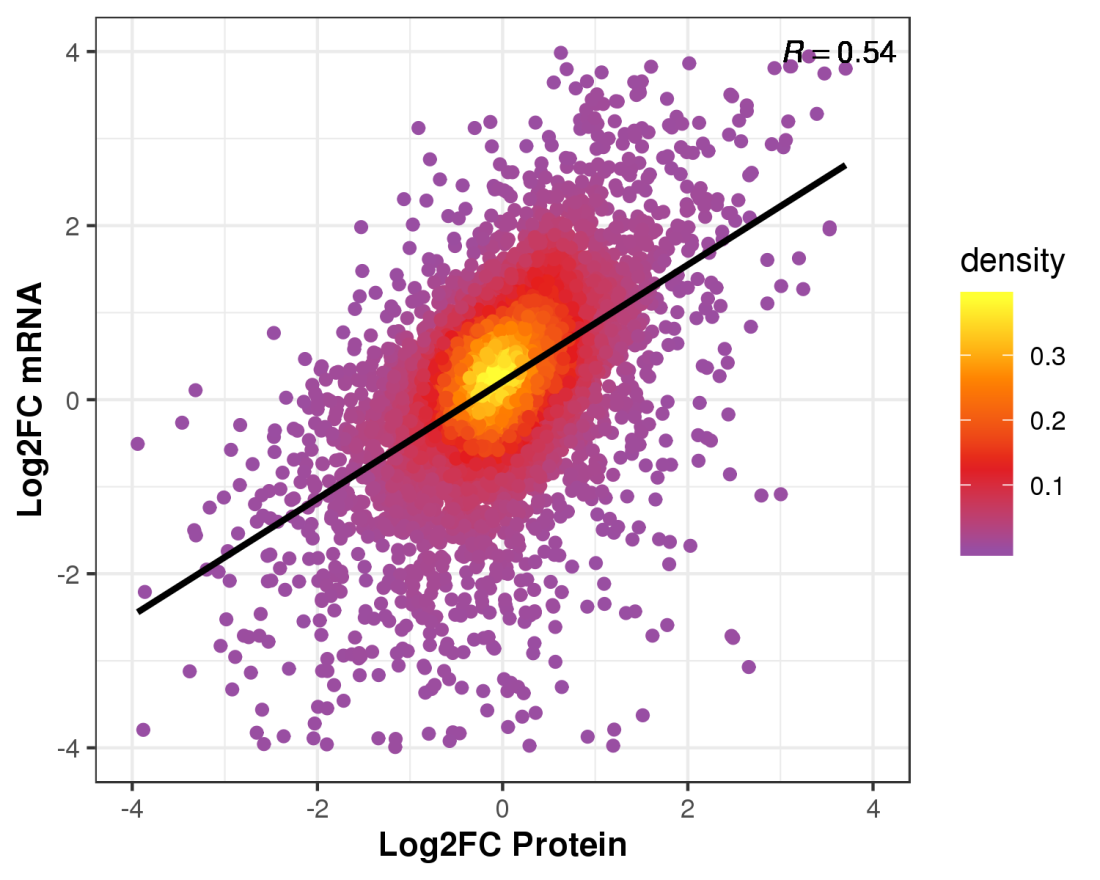


**Figure S5.** A scatter plot of the transcript and its corresponding protein expression. Note: In the figure, the horizontal axis is protein expression level, and the vertical axis is transcript expression level.The color of a point represents its density. R, correlation coefficient of all quantified proteins and their corresponding mRNAs


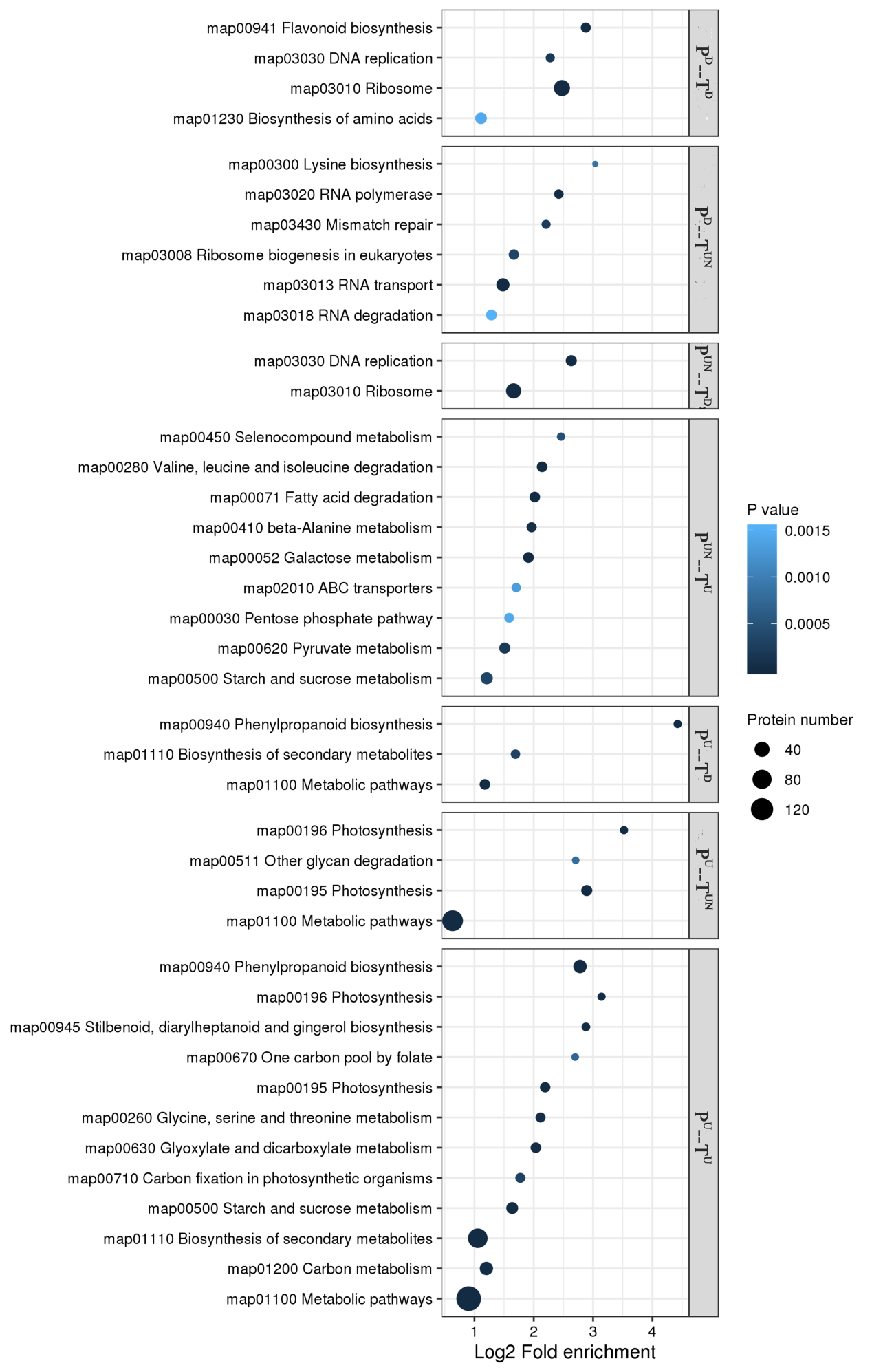


**Figure S6.** KEGG pathway. Note: The enrichment results of different classifications were combined, and the functional classification with top40 significant enrichment (P <0.05) was screened out, and the results were displayed in the form of bubble chart.In the figure, Fold enrichment after Log2 log conversion is shown on the horizontal axis, while functional classification is shown on the vertical axis. The bubble size represents the number of proteins, and the bubble color represents the enrichment significance Pvalue.type:Description of the differential expression of protein and transcript.P^D^--T^D^:protein down-gene down; P^D^--T^UN^: protein down-gene unchange; P^UN^--T^D^:protein unchange-gene down;P^UN^--T^U^:protein unchange-gene up ;P^U^--T^D^:protein up-gene down;P^U^--T^UN^:protein up-gene unchange;P^U^--T^U^:protein up-gene up.


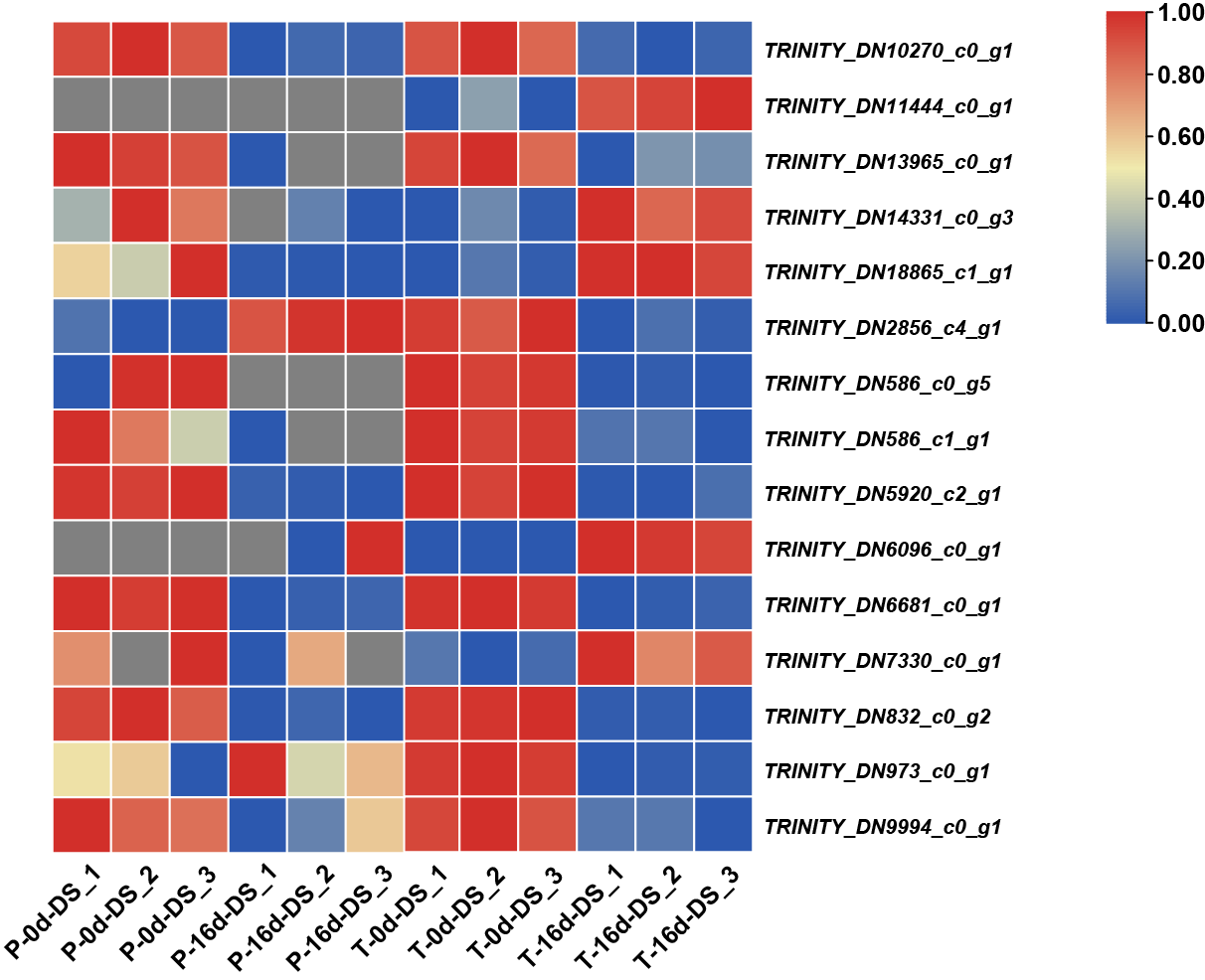


**Figure S7**. Heatmap of DEGs and DEPs related to HSPs in Nanmu. note:P-0d-DS: This color represents the change in the expression of DEGs/DEPs. Blue to red represent the expression levels of DEGs/DEPs from low to high.Non-significant genes/proteins are shown in grey.P-0d-DS, proteome-0-daydrought-stressed;P-16d-DS,proteome-16-daydrought-stressed;T-0d-DS,transcriptome16-day drought-stressed . Numbers of type "_1" represent the number of repetitions

**Table S1.** A detailed list of primer sequences used in this study.

| Gene number | gene symbol | Primer name | sequence(5’-3’) | Primer TM value(℃) | Product length(bp) |
| --- | --- | --- | --- | --- | --- |
| TRINITY_DN3407_c1_g3 | PIP2-7 | ZD 1 F | TGCATCATCCCTACAACCGT | 59.09 | 126 |
|  |  | ZD 1 R | TGGCGGAGAAGACAGTGTAG | 59.11 |  |
| TRINITY_DN4800_c0_g1 | CAT1 | ZD 9 F | CGAGGAGGAATGGGGAACTT | 59.09 | 146 |
|  |  | ZD 9 R | CTTCCACCAAATGCACCCAA | 58.95 |  |
| TRINITY_DN1523_c0_g1 |  | ZD 12 F1 | AAGTGGAAGGAGGTGTCGAG | 59.03 | 109 |
|  |  | ZD 12 R1 | TCTTCTCCCACCACTCGAAC | 59.03 |  |
| TRINITY_DN498_c1_g1 |  | ZD 13 F | CCTCAGACCTTCCGTTCCAA | 59.31 | 102 |
|  |  | ZD 13 R | ATGATCTGGGTGCTCTCCTG | 58.87 |  |
| TRINITY_DN5434_c0_g1 | 4CLL9 | ZD 20 F | GTACGGTGGATGGAGAGAGG | 58.97 | 106 |
|  |  | ZD 20 R | TCTCAATTCCTCGACCGCAT | 59.18 |  |
| TRINITY_DN1443_c2_g2 |  | ZD 14 F | GGCCTTGGACCATTTTCGTT | 59.03 | 106 |
|  |  | ZD 14 R | CCCAACTTAGCATCAAGGGC | 58.89 |  |
| TRINITY_DN16577_c0_g1 | PED1 | ZD 22 F | CGTAGAATGCTGCCATCCTG | 58.78 | 148 |
|  |  | ZD 22 R | CATGCTGATGACCTTCTCGC | 59.06 |  |

**Table S2.** The statistical results compared with the reference sequence.

| #SampleID | ReadSum | BaseSum | GC(%) | Q20(%) | Q30(%) |
| --- | --- | --- | --- | --- | --- |
| 0d-DS_1 | 20,861,158 | 6,258,347,400 | 45.25% | 98.08% | 94.38% |
| 0d-DS_2 | 21,519,755 | 6,455,926,500 | 45.21% | 98.40% | 95.18% |
| 0d-DS_3 | 20,999,948 | 6,299,984,400 | 45.19% | 98.18% | 94.65% |
| 16-DS_1 | 24,177,356 | 7,253,206,800 | 46.21% | 98.22% | 94.71% |
| 16-DS_2 | 22,285,306 | 6,685,591,800 | 46.16% | 98.18% | 94.65% |
| 16-DS_3 | 25,436,599 | 7,630,979,700 | 46.18% | 98.17% | 94.69% |

Note: SampleID: sample name of the sample information sheet; ReadSum: total number of pair-end Reads in clean Data; BaseSum: total bases in Clean Data; GC: Clean Data GC content, that is, G and C bases in Clean Data account for total bases Percentage of bases; ≥Q30%: The percentage of bases whose Clean Data quality value is greater than or equal to 30. 0d-DS, 0-day drought-stressed; 16d-DS, drought-stressed. “_1” represents the number of repetitions

**Table S3.** Statistical table of assembly results.

| # | Unigenes | Transcripts |
| --- | --- | --- |
| Total_seq_num | 60250 | 68716 |
| Total_seq_len | 44409579 | 57924344 |
| Max_len | 17779 | 17779 |
| Min_len | 201 | 201 |
| Average_len | 737 | 842 |
| Percent_GC | 42.52% | 42.73% |
| N40 | 2305 | 2374 |
| N50 | 1878 | 1974 |
| N60 | 1466 | 1602 |
| N70 | 956 | 1177 |
| N80 | 274 | 518 |
| N90 | 243 | 251 |

Note: Total_seq_num: total number of sequences;Total_seq_len: sum of the lengths of all sequences;Max_len: maximum length (bp);Min_len: minimum length (bp);Average_len: average length of all sequences assembled (BP);Percent_GC: total number of bases in GC as a percentage of total bases;N50: Sequence assembled transcripts from long to short in terms of length, and when the cumulative length of transcripts reaches 50% of the total length, the corresponding length of transcripts, and so on.

**Table S4. Overlap of DEGs and DEPs in nanmu (genotype)**

| Classification | Transcription.ID | Protein.description | gene symbol | log2FC.x | log2FC.y | Type(protein-transcript) | |
| --- | --- | --- | --- | --- | --- | --- | --- |
| Antioxdase | TRINITY_DN12406_c0_g2 | Peroxidase 12 | POD12 | 0.305329 | 1.576951 | unchange-up | |
|  | TRINITY_DN12429_c0_g1 | Peroxidase 52 | POD52 | -0.06567 | 1.847422 | unchange-up | |
|  | TRINITY_DN941_c1_g1 | Peroxidase 39 | POD39 | NA | 3.75215 | unchange-up | |
|  | TRINITY_DN20_c0_g1 | Monodehydroascorbate reductase | MDHAR | 0.190678 | 1.027913 | unchange-up | |
|  | TRINITY_DN3465_c0_g1 | Monodehydroascorbate reductase 4, peroxisomal | MDHAR4 | 0.453439 | 1.305985 | unchange-up | |
|  | TRINITY_DN19728_c0_g1 | Cationic peroxidase 1 | CAT1 | NA | 2.293057 | unchange-up | |
|  | TRINITY_DN17181_c0_g1 | Peroxidase 4 | POD4 | 1.274351 | 2.653487 | up-up |  |
|  | TRINITY_DN21461_c0_g1 | Peroxidase 5 | POD5 | 1.139077 | 1.84537 | up-up |  |
|  | TRINITY_DN29629_c0_g1 | Peroxidase 72 | POD72 | 1.195096 | 7.869462 | up-up |  |
|  | TRINITY_DN3054_c0_g1 | Peroxidase 64 | POD64 | 3.475163 | 3.747576 | up-up |  |
|  | TRINITY_DN7850_c0_g1 | Superoxide dismutase [Cu-Zn] | SODCC | 0.620493 | -0.26611 | up-unchange | |
|  | TRINITY_DN9758_c0_g1 | Superoxide dismutase [Cu-Zn] | SODCP | 1.108491 | -0.34041 | up-unchange | |
|  | TRINITY_DN4066_c1_g1 | Glutathione S-transferase F9 | GSTF9 | -1.3485 | -0.81263 | down-unchange | |
|  | TRINITY_DN2656_c0_g1 | Glutathione S-transferase F13 | GSTF13 | -1.31366 | -1.19032 | unchange-down | |
|  | TRINITY_DN6768_c0_g1 | Glutathione S-transferase U17 | GSTU17 | -1.23447 | -0.15451 | down-unchange | |
| ABA biosynthesis&/signaling transduction | TRINITY_DN10357_c0_g1 | Probable protein phosphatase 39 | PP2C 39 | NA | -1.05578 | unchange-down | |
|  | TRINITY_DN1071_c0_g1 | Serine/threonine-protein kinase | Snrk2 | 2.375735 | 1.842591 | up-up |  |
|  | TRINITY_DN1140_c0_g1 | Indole-3-acetaldehyde oxidase | AAO3 | 0.538041 | 1.007024 | unchange-up | |
|  | TRINITY_DN1412_c0_g1 | Protein phosphatase 2C 70 | PP2C 70 | NA | 1.236681 | unchange-up | |
|  | TRINITY_DN1443_c2_g2 | Zeaxanthin epoxidase | ZEP | 0.854474 | 1.790181 | up-up |  |
|  | TRINITY_DN2303_c0_g1 | Secoisolariciresinol dehydrogenase (Fragment) | ABA2 | 0.064331 | 2.078912 | unchange-up | |
|  | TRINITY_DN2625_c1_g1 | Probable protein phosphatase 2C 44 | PP2C 44 | 0.606821 | -0.05282 | up-unchange | |
|  | TRINITY_DN328_c0_g1 | Serine/threonine-protein kinase | Snrk3 | 1.040121 | 1.05858 | up-up |  |
|  | TRINITY_DN3799_c0_g1 | Probable protein phosphatase 2C 9 | PP2C 9 | 0.55454 | 1.155405 | unchange-up | |
|  | TRINITY_DN4061_c0_g1 | Probable protein phosphatase 2C 10 | PP2C 10 | 0.467801 | 1.002135 | unchange-up | |
|  | TRINITY_DN4083_c0_g1 | Probable protein phosphatase 2C 15 | PP2C 15 | -0.27526 | -1.12856 | unchange-down | |
|  | TRINITY_DN4488_c0_g2 | Probable protein phosphatase 2C 66 | PP2C 66 | 1.665848 | 1.158162 | up-up |  |
|  | TRINITY_DN601_c0_g1 | Probable carotenoid cleavage dioxygenase 4 | NCED | 0.253263 | 1.250704 | unchange-up | |
|  | TRINITY_DN6167_c0_g1 | Probable protein phosphatase 2C | BIPP2C1 | 1.301295 | 0.987943 | up-unchange | |
|  | TRINITY_DN6468_c0_g1 | Protein phosphatase 2C 57 | PP2C 57 | 0.857185 | 2.505379 | up-up |  |
|  | TRINITY_DN6468_c0_g2 | Protein phosphatase 2C 57 | PP2C 57 | NA | 1.327097 | unchange-up | |
|  | TRINITY_DN709_c0_g1 | Protein phosphatase 2C 56 | PP2C 56 | -0.152 | 1.1579 | unchange-up | |
|  | TRINITY_DN9446_c0_g1 | MLP-like protein 423 | PYL | -0.63218 | -3.11741 | down-down | |
|  | TRINITY_DN9737_c0_g2 | Protein phosphatase 2C 16 | PP2C 16 | 0.280362 | 1.130226 | unchange-up | |
|  | TRINITY_DN19547_c0_g1 | Calcium-binding protein CBP | CBP | -0.58879 | -1.08967 | down-down | |
| Aquaporin | TRINITY_DN3407_c1_g2 | Aquaporin PIP2-7 | PIP2-7 | -1.38836 | -0.38217 | down-unchange | |
|  | TRINITY_DN3407_c2_g1 | Aquaporin PIP2-4 | PIP2-4 | -0.35328 | 1.018524 | unchange-up | |
|  | TRINITY_DN625_c2_g1 | Probable aquaporin PIP1-4 | PIP1.4 | -0.79436 | 0.328907 | down-unchange | |
|  | TRINITY_DN14832_c0_g1 | Aquaporin TIP2-1 | TIP2-1 | 4.297677 | Inf | up-down |  |
|  | TRINITY_DN3024_c0_g1 | Aquaporin TIP1-3 | TIP1-3 | 1.413833 | Inf | unchange-down | |
|  | TRINITY_DN7917_c0_g1 | Aquaporin TIP1-1 | TIP1-1 | NA | Inf | unchange-down | |
| SAbiosynthesis&signaling | TRINITY_DN9097_c0_g1 | T-complex protein 1 subunit gamma | CCT3 | -0.87303 | -0.66785 | down-unchange | |
|  | TRINITY_DN6959_c0_g1 | Protein STRICTOSIDINE SYNTHASE-LIKE 6 | SSL6 | 0.983386 | -0.12846 |  |  |
|  | TRINITY_DN4598_c0_g2 | Putative disease resistance protein At1g50180 | // | -1.15972 | -3.99115 | down-down | |
|  | TRINITY_DN214_c0_g1 | 4-hydroxy-3-methylbut-2-en-1-yl diphosphate synthase (ferredoxin), chloroplastic | ISPG | -0.78787 | 0.945483 | down-unchange | |
|  | TRINITY_DN13523_c0_g1 | Serine/threonine-protein kinase/endoribonuclease IRE1a | IRE1A | -1.0526 | 0.201584 | down-unchange | |
|  | TRINITY_DN8558_c0_g1 | Mitogen-activated protein kinase 4 | MPK4 | 0.888695 | 1.634006 | up-up |  |
|  | TRINITY_DN6032_c0_g1 | Caffeic acid 3-O-methyltransferase 1 | OMT1 | 1.504112 | 3.119474 | up-up |  |
|  | TRINITY_DN4698_c2_g1 | Caffeic acid 3-O-methyltransferase | COMT1 | 2.370666 | 1.929538 | up-up |  |
|  | TRINITY_DN3147_c6_g1 | Transcription factor TGA4 | TGA4 | 0.676358 | 1.453269 | up-up |  |
|  | TRINITY_DN17833_c0_g1 | Caffeic acid 3-O-methyltransferase 1 | HOMT1 | 1.615981 | 1.616353 | up-up |  |
|  | TRINITY_DN833_c1_g1 | Caffeic acid 3-O-methyltransferase 1 | OMT1 | 0.748891 | 0.829714 | up-unchange | |
|  | TRINITY_DN6032_c0_g2 | Caffeic acid 3-O-methyltransferase 1 | HOMT1 | 1.238237 | 0.46931 | up-unchange | |
|  | TRINITY_DN10_c0_g1 | Linoleate 13S-lipoxygenase 2-1, chloroplastic | LOX2.1 | -0.66497 | 1.228749 | down-up |  |
|  | TRINITY_DN10387_c0_g1 | Probable linoleate 9S-lipoxygenase 5 | LOX1.5 | -0.58727 | -0.47566 | down-unchange | |
|  | TRINITY_DN461_c0_g1 | Linoleate 13S-lipoxygenase 3-1, chloroplastic | LOX3.1 | 0.451646 | -1.74715 | unchange-down | |
| JA biosynthesis | TRINITY_DN12465_c0_g1 | 12-oxophytodienoate reductase 2 | OPR2 | NA | -1.14781 | unchange-down | |
|  | TRINITY_DN15667_c0_g1 | Allene oxide synthase 1, chloroplastic | AOS1 | -0.71715 | -2.49421 | unchange-down | |
|  | TRINITY_DN27878_c0_g2 | Jasmonate O-methyltransferase | JMT | -0.42353 | -2.90172 | unchange-down | |
|  | TRINITY_DN10387_c0_g1 | Probable linoleate 9S-lipoxygenase 5 | LOX1.5 | -0.58727 | -0.47566 | down-unchange | |
|  | TRINITY_DN15667_c0_g1 | Allene oxide synthase 1 | AOS1 | -0.71715 | -2.49421 | unchange-down | |
|  | TRINITY_DN2461_c0_g2 | 12-oxophytodienoate reductase 2 | OPR2 | 0.888227 | 0.166019 | up-unchange | |
|  | TRINITY_DN32121_c0_g1 | Peroxisomal acyl-coenzyme A oxidase 1 | ACX1 | 0.255803 | 1.56894 | unchange-up | |
|  | TRINITY_DN4579_c0_g1 | Acyl-coenzyme A oxidase 3, peroxisomal | ACX3 | 0.620868 | 0.163635 | up-unchange | |
|  | TRINITY_DN461_c0_g1 | Linoleate 13S-lipoxygenase 3-1 | LOX3.1 | 0.451646 | -1.74715 | unchange-down | |
|  | TRINITY_DN5086_c0_g1 | Allene oxide synthase 1 | AOS1 | 0.805045 | 0.108842 | up-unchange | |
|  | TRINITY_DN512_c1_g1 | Lipoxygenase 6 | LOX6 | 0.866235 | 0.316871 | up-unchange | |
|  |  |  |  |  |  |  | |
| Phenylpropanoid biosynthesis | TRINITY_DN10934_c0_g1 | Probable cinnamyl alcohol dehydrogenase 1 | CAD1 | 0.791689 | 1.046159 | up-up |  |
|  | TRINITY_DN11825_c0_g1 | Cytochrome P450 CYP73A100 | C4H | NA | -2.27774 | unchange-down | |
|  | TRINITY_DN13809_c0_g1 | (S)-scoulerine 9-O-methyltransferase | COMT1 | 2.230541 | 1.687369 | up-up |  |
|  | TRINITY_DN1411_c0_g1 | Shikimate O-hydroxycinnamoyltransferase | HCT | 1.166715 | 0.57904 | up-unchange | |
|  | TRINITY_DN17833_c0_g1 | Caffeic acid 3-O-methyltransferase 1 | COMT1 | 1.615981 | 1.616353 | up-up |  |
|  | TRINITY_DN190_c0_g1 | Probable mannitol dehydrogenase | CAD | -1.39403 | -0.89863 | down-unchange | |
|  | TRINITY_DN2557_c0_g1 | Probable cinnamyl alcohol dehydrogenase 6 | CAD6 | 0.751635 | -0.89698 | up-unchange | |
|  | TRINITY_DN2851_c0_g1 | Caffeoyl-CoA O-methyltransferase | CCoAOMT | 2.137208 | 2.430623 | up-up |  |
|  | TRINITY_DN32732_c0_g2 | Cinnamoyl-CoA reductase 1 | CCR1 | 1.619976 | 1.573782 | up-up |  |
|  | TRINITY_DN3333_c0_g1 | Phenylalanine ammonia-lyase | PAL | -7.96578 | -4.54443 | down-down | |
|  | TRINITY_DN3333_c0_g2 | Phenylalanine ammonia-lyase | PAL | -8.82828 | -5.95838 | down-down | |
|  | TRINITY_DN3333_c2_g1 | Phenylalanine ammonia-lyase 3 | PAL3 | 0.985136 | 1.900853 | up-up |  |
|  | TRINITY_DN3627_c0_g1 | Shikimate O-hydroxycinnamoyltransferase | HCT | 3.393018 | 3.284235 | up-up |  |
|  | TRINITY_DN3627_c0_g2 | Shikimate O-hydroxycinnamoyltransferase | HCT | 2.313188 | 2.236541 | up-up |  |
|  | TRINITY_DN4698_c2_g1 | Caffeic acid 3-O-methyltransferase | COMT1 | 2.370666 | 1.929538 | up-up |  |
|  | TRINITY_DN5224_c0_g1 | Probable caffeoyl-CoA O-methyltransferase | CCoAOMT | 0.653977 | 0.788762 | up-unchange | |
|  | TRINITY_DN5224_c0_g3 | Probable caffeoyl-CoA O-methyltransferase At4g26220 | CCoAOMT | 1.988048 | 2.84457 | up-up |  |
|  | TRINITY_DN5826_c0_g1 | Probable cinnamyl alcohol dehydrogenase 1 | CAD1 | 0.80331 | 1.553177 | up-up |  |
|  | TRINITY_DN5826_c0_g2 | Probable cinnamyl alcohol dehydrogenase 1 | CAD1 | 2.101751 | 2.359267 | up-up |  |
|  | TRINITY_DN5941_c0_g1 | 4-coumarate--CoA ligase 2 | 4CL2 | 2.444932 | 3.044929 | up-up |  |
|  | TRINITY_DN6032_c0_g1 | Caffeic acid 3-O-methyltransferase 1 | COMT1 | 1.504112 | 3.119474 | up-up |  |
|  | TRINITY_DN6032_c0_g2 | Caffeic acid 3-O-methyltransferase 1 | COMT1 | 1.238237 | 0.46931 | up-unchange | |
|  | TRINITY_DN624_c0_g1 | Stemmadenine O-acetyltransferase | HCT | 1.431142 | 3.020966 | up-up |  |
|  | TRINITY_DN6563_c0_g1 | 4-coumarate--CoA ligase 2 | 4CL2 | -7.96578 | -3.64122 | down-down | |
|  | TRINITY_DN833_c1_g1 | Caffeic acid 3-O-methyltransferase 1 | COMT1 | 0.748891 | 0.829714 | up-unchange | |
| Flavonoid biosynthesis | TRINITY_DN10140_c0_g1 | Leucoanthocyanidin dioxygenase | ANS | -4.77596 | -5.07912 | down-down | |
|  | TRINITY_DN12674_c0_g1 | Flavonoid 3'-monooxygenase | F3'H | -3.37932 | -3.12148 | down-down | |
|  | TRINITY_DN241_c3_g1 | Protein DMR6-LIKE OXYGENASE 2 | FLS | 1.068533 | 3.760594 | up-up |  |
|  | TRINITY_DN28725_c0_g1 | Probable chalcone--flavonone isomerase 3 | CHI3 | -2.30757 | -3.09108 | down-down | |
|  | TRINITY_DN2990_c0_g1 | Chalcone synthase 1 | CHS1 | NA | -6.46857 | unchange-down | |
|  | TRINITY_DN4906_c0_g1 | Flavonol synthase/flavanone 3-hydroxylase | FLS | -3.02562 | -4.02269 | down-down | |
|  | TRINITY_DN5209_c0_g1 | Chalcone synthase | CHS | NA | -6.77769 | unchange-down | |
|  | TRINITY_DN5789_c0_g1 | Flavanone 3-dioxygenase | F3H | -2.74178 | -2.72908 | down-down | |
|  | TRINITY_DN5789_c0_g2 | Naringenin,2-oxoglutarate 3-dioxygenase (Fragment) | F3H | -2.71881 | -3.13643 | down-down | |
|  | TRINITY_DN6146_c1_g1 | Putative anthocyanidin reductase | DFR | 1.223731 | 3.429459 | up-up |  |
|  | TRINITY_DN6653_c0_g1 | Chalcone synthase 1 | CHS1 | -3.88044 | -3.79352 | down-down | |
|  | TRINITY_DN7336_c0_g1 | Bifunctional dihydroflavonol 4-reductase/flavanone 4-reductase | DFR | -2.8879 | -2.956 | down-down | |
|  | TRINITY_DN8171_c0_g3 | Flavonol synthase/flavanone 3-hydroxylase | FLS | -1.85465 | -2.25509 | down-down | |
| heat shock protein | TRINITY_DN10270_c0_g1 | Heat shock 70 kDa protein, mitochondrial | HSP | -3.941198646 | -0.507905712 | down-unchange | |
|  | TRINITY_DN11444_c0_g1 | 17.3 kDa class I heat shock protein | HSP17.3-B | NA | 2.029159869 | unchange-up | |
|  | TRINITY_DN13965_c0_g1 | Heat shock protein 83 OS=Ipomoea nil | HSP83A | -3.196276993 | -1.952347541 | unchange-down | |
|  | TRINITY_DN14331_c0_g3 | Heat shock cognate 70 kDa protein 2 | HSC-2 | -0.969734557 | 2.012220825 | unchange-up | |
|  | TRINITY_DN18865_c1_g1 | Heat shock cognate 70 kDa protein | HSP70 | -0.553002759 | 4.187474727 | unchange-up | |
|  | TRINITY_DN586_c0_g5 | Heat shock protein 90-6 | HSP90-6 | NA | -2.725350763 | unchange-down | |
|  | TRINITY_DN586_c1_g1 | Heat shock cognate protein 80 | HSC80 | -0.646789644 | -1.019004345 | unchange-down | |
|  | TRINITY_DN5920_c2_g1 | Activator of 90 kDa heat shock protein ATPase homolog 2 | Ahsa2 | -1.336792028 | -1.112749947 | down-down | |
|  | TRINITY_DN6096_c0_g1 | Heat shock cognate 70 kDa protein 2 | HSP70 | NA | 8.523775856 | unchange-up | |
|  | TRINITY_DN6681_c0_g1 | Small heat shock protein | HSP23 | -2.498994047 | -2.078129453 | down-down | |
|  | TRINITY_DN7330_c0_g1 | Heat shock protein 90-5 | HSP90-5 | -0.376320392 | 1.441306266 | unchange-up | |
|  | TRINITY_DN832_c0_g2 | Heat shock 70 kDa protein 17 | HSP70-17 | -0.654490076 | -0.852572524 | down-unchange | |
|  | TRINITY_DN973_c0_g1 | 19.0 kDa class II heat shock protein | HSP19.0 | 0.074916402 | -2.514387439 | unchange-down | |
|  | TRINITY_DN9994_c0_g1 | 15.7 kDa heat shock protein | HSP15.7 | -0.699700526 | -1.54666827 | down-down | |
|  | TRINITY_DN2856_c4_g1 | Late embryogenesis abundant protein Lea14-A | LEA | 0.915941506 | 0.023420629 | up-unchange | |
| photosynthesis and photosynthesis-antenna proteins | TRINITY_DN375_c0_g1 | Photosystem I reaction center subunit III | PSAF | 1.159758 | 0.898796 | up-unchange | |
|  | TRINITY_DN4408_c0_g1 | Plastocyanin | PETE | 0.751721 | 1.466971 | up-up |  |
|  | TRINITY_DN47864_c0_g1 | Photosystem II protein D1 | PSBA | 1.002162 | 1.783648 | up-up |  |
|  | TRINITY_DN4824_c0_g1 | Cytochrome b6 | PETB | 1.441855 | 0.072363 | up-unchange | |
|  | TRINITY_DN5004_c0_g1 | Plastocyanin, chloroplastic | PETE | 1.023823 | 0.597309 | up-unchange | |
|  | TRINITY_DN51307_c0_g1 | Photosystem II reaction center PSB28 protein | PSB28 | 0.696172 | 0.10254 | up-unchange | |
|  | TRINITY_DN523_c0_g1 | Cytochrome b6-f complex iron-sulfur subunit | PETC | 0.593688 | 1.597038 | up-up |  |
|  | TRINITY_DN523_c0_g2 | Cytochrome b6-f complex iron-sulfur subunit | PETC | 0.982948 | 1.228236 | up-up |  |
|  | TRINITY_DN1647_c2_g3 | ATP synthase delta chain, chloroplastic | ATPD | 0.923263 | 0.754649 | up-unchange | |
|  | TRINITY_DN10938_c1_g2 | Photosystem II CP43 reaction center protein | PSBC | 1.013284 | 0.50891 | up-unchange | |
|  | TRINITY_DN5303_c1_g1 | Oxygen-evolving enhancer protein 1 | PSBO | 1.026658 | 1.123951 | up-up |  |
|  | TRINITY_DN6222_c0_g1 | Photosynthetic NDH subunit of lumenal location 1 | PNSL1 | 0.618802 | 1.202475 | up-up |  |
|  | TRINITY_DN21618_c0_g1 | PsbP-like protein 1 | PPL1 | 0.881116 | 0.591431 | up-unchange | |
|  | TRINITY_DN1645_c1_g1 | Oxygen-evolving enhancer protein 3 | PSBQ | 1.829038 | 1.32368 | up-up |  |
|  | TRINITY_DN2083_c1_g1 | Photosystem II repair protein PSB27-H1 | PSB27-1 | 1.244217 | 0.079986 | up-unchange | |
|  | TRINITY_DN671_c0_g2 | Photosystem I reaction center subunit II | PSAD | 1.338567 | 0.982895 | up-unchange | |
|  | TRINITY_DN2659_c0_g1 | Photosystem I reaction center subunit IV A | PSAEA | 1.396927 | 0.148881 | up-unchange | |
|  | TRINITY_DN2432_c1_g1 | Photosystem I reaction center subunit IV | PSAE | 2.253112 | 1.344599 | up-up |  |
|  | TRINITY_DN3397_c1_g1 | Photosystem I reaction center subunit V | PSAG | 1.536152 | 1.422118 | up-up |  |
|  | TRINITY_DN14652_c0_g1 | Rop guanine nucleotide exchange factor 12 | ROPGEF12 | 1.420671 | 0.865439 | up-unchange | |
|  | TRINITY_DN1094_c0_g1 | Ferredoxin-3 | PETF | -1.0908 | -2.32349 | down-down | |
|  | TRINITY_DN1647_c2_g3 | ATP synthase delta chain | ATPD | 0.923263 | 0.754649 | up-unchange | |
|  | TRINITY_DN34597_c0_g1 | ATP synthase subunit b' | ATPG | 0.91311 | 1.872571 | up-up |  |
|  | TRINITY_DN7126_c0_g2 | Oxygen-evolving enhancer protein 2V=1 | PSBP | 1.160275 | 1.32943 | up-up |  |
|  | TRINITY_DN896_c0_g1 | Photosystem I reaction center subunit N | PSAN | 1.462628 | 0.730407 | up-unchange | |
|  | TRINITY_DN9695_c0_g2 | Tetratricopeptide repeat domain-containing protein PYG7 | PYG7 | 0.680864 | 0.059348 | up-unchange | |
|  | TRINITY_DN874_c0_g1 | Photosystem II 10 kDa polypeptide | PSBR | 1.305562 | 0.940542 | up-unchange | |
|  | TRINITY_DN694_c0_g1 | Photosystem I reaction center subunit XI | PSAL | 1.501006 | 1.09994 | up-up | |
|  | TRINITY_DN183_c0_g1 | Chlorophyll a-b binding protein 6A | LHCA1 | 1.427123 | 1.457805 | up-up |  |
|  | TRINITY_DN1928_c0_g1 | Chlorophyll a-b binding protein 3 | CAB3 | 1.378345 | 0.657217 | up-unchange | |
|  | TRINITY_DN2020_c0_g1 | Chlorophyll a-b binding protein 8 | LHCA3 | 1.357721 | 1.31876 | up-up |  |
|  | TRINITY_DN3573_c0_g1 | Chlorophyll a-b binding protein CP26 | LHCB5 | 1.297896 | 1.337869 | up-up |  |
|  | TRINITY_DN3685_c0_g1 | Ribonucleoside-diphosphate reductase small chain | LHCA2 | 1.483003 | 0.866219 | up-unchange | |
|  | TRINITY_DN3812_c0_g1 | Chlorophyll a-b binding protein 13 | LHCB3 | 1.336626 | 1.584931 | up-up |  |
|  | TRINITY_DN4039_c0_g1 | Chlorophyll a-b binding protein 151 | CAB-151 | 1.179193 | 0.900299 | up-unchange | |
|  | TRINITY_DN5224_c0_g2 | Chlorophyll a-b binding protein CP29.2 | LHCB4.2 | 0.911116 | 0.98278 | up-unchange | |
|  | TRINITY_DN575_c0_g1 | Chlorophyll a-b binding protein CP24 10A | CAP10A | 1.427553 | 1.248443 | up-up |  |
|  | TRINITY_DN5908_c0_g1 | Chlorophyll a-b binding protein 3C | CAB3C | 0.716157 | 1.04597 | up-up |  |
|  | TRINITY_DN6554_c0_g1 | Chlorophyll a-b binding protein 4 | LHCA4 | 1.091124 | 0.954103 | up-unchange | |
|  | TRINITY_DN76454_c0_g1 | Chlorophyll a-b binding protein CP29.2 | LHCB4.2 | 1.31342 | 0.116228 | up-unchange | |
| Chlorophyll synthesis and metabolism | TRINITY_DN7357_c1_g2 | Pheophorbide a oxygenase | PAO | 0.692963 | 1.434607 | up-up |  |
|  | TRINITY_DN9850_c0_g1 | Magnesium-chelatase subunit ChlH | CHLH | -0.80163 | 1.49085 | down-up |  |
|  | TRINITY_DN10969_c0_g1 | Magnesium-chelatase subunit ChlD | CHID | -0.60933 | 0.733227 | down-unchange | |
| Metabolic pathway | TRINITY_DN1031_c1_g1 | Glutamate decarboxylase 1 | GAD1 | 1.351854 | 2.609716 | up-up |  |
|  | TRINITY_DN1660_c0_g1 | Basic 30 kDa endochitinase | CHI9 | 1.293253 | 1.182563 | up-up |  |
|  | TRINITY_DN17298_c0_g1 | Probable glutathione S-transferase parA | PARA | 0.599984 | 1.163144 | up-up |  |
|  | TRINITY_DN21461_c0_g1 | Peroxidase 5 OS=Vitis vinifera | GSVIVT00037159001 | 1.139077 | 1.84537 | up-up |  |
|  | TRINITY_DN4800_c0_g1 | Catalase isozyme 1 | CAT1 | 2.003566 | 1.71106 | up-up |  |
|  | TRINITY_DN4800_c0_g1 | Catalase isozyme 1 | CAT1 | 0.498047 | 1.71106 | unchange-up | |
|  | TRINITY_DN579_c0_g1 | Long chain acyl-CoA synthetase 1 | LACS1 | 1.065159 | 1.888213 | up-up |  |
|  | TRINITY_DN7634_c0_g1 | Phosphoribulokinase | // | 0.589284 | 1.302489 | up-up |  |
| Kelvin pathway | TRINITY_DN629_c0_g2 | Glyceraldehyde-3-phosphate dehydrogenase GAPCP1 | GAPCP1 | -1.82674 | -0.45979 | down-unchange | |
|  | TRINITY_DN629_c0_g3 | Glyceraldehyde-3-phosphate dehydrogenase GAPCP1 | GAPCP1 | -2.41427 | -4.44921 | down-down | |
|  | TRINITY_DN5655_c0_g1 | Glyceraldehyde-3-phosphate dehydrogenase GAPCP2 | GAPCP2 | -1.45482 | -1.5748 | down-down | |
| Soluble sugar | TRINITY_DN3572_c2_g1 | Probable alpha,alpha-trehalose-phosphate synthase | TPS9 | 1.355242 | 1.175244 | up-up |  |

Note:log2FC.x,The value of protein expression ratio after Log2 logarithmic transformation;log2FC.y,The value of transcript ratio after Log2 logarithmic rotation, "//" means no gene name
